# Pre-clinical study of IRDye800CW-nimotuzumab formulation, stability, pharmacokinetics, and safety

**DOI:** 10.1186/s12885-021-08003-3

**Published:** 2021-03-12

**Authors:** Wendy Bernhard, Kris Barreto, Ayman El-Sayed, Carolina Gonzalez, Raja Solomon Viswas, Darien Toledo, Angel Casaco, John DeCoteau, Humphrey Fonge, Clarence Ronald Geyer

**Affiliations:** 1 Department of Pathology and Laboratory Medicine, College of Medicine, University of Saskatchewan, Saskatoon, SK Canada; 2 Department of Medical Imaging, College of Medicine, University of Saskatchewan, Saskatoon, SK Canada; 3 Center of Molecular Immunology, Havana, Cuba; 4 Department of Medical Imaging, Royal University Hospital, Saskatoon, SK Canada

**Keywords:** Nimotuzumab, EGFR, Near-infrared fluorescence imaging, IRDye800CW, Image-guided surgery, Investigator’s brochure

## Abstract

**Background:**

Epidermal growth factor receptor (EGFR) is a target for cancer therapy as it is overexpressed in a wide variety of cancers. Therapeutic antibodies that bind EGFR are being evaluated in clinical trials as imaging agents for positron emission tomography and image-guided surgery. However, some of these antibodies have safety concerns such as infusion reactions, limiting their use in imaging applications. Nimotuzumab is a therapeutic monoclonal antibody that is specific for EGFR and has been used as a therapy in a number of countries.

**Methods:**

Formulation of IRDye800CW-nimotuzumab for a clinical trial application was prepared. The physical, chemical, and pharmaceutical properties were tested to develop the specifications to determine stability of the product. The acute and delayed toxicities were tested and IRDye800CW-nimotuzumab was determined to be non-toxic. Non-compartmental pharmacokinetics analysis was used to determine the half-life of IRDye800CW-nimotuzumab.

**Results:**

IRDye800CW-nimotuzumab was determined to be non-toxic from the acute and delayed toxicity study. The half-life of IRDye800CW-nimotuzumab was determined to be 38 ± 1.5 h. A bi-exponential analysis was also used which gave a t_1/2_ alpha of 1.5 h and t_1/2_ beta of 40.8 h.

**Conclusions:**

Here, we show preclinical studies demonstrating that nimotuzumab conjugated to IRDye800CW is safe and does not exhibit toxicities commonly associated with EGFR targeting antibodies.

**Supplementary Information:**

The online version contains supplementary material available at 10.1186/s12885-021-08003-3.

## Background

**Fig. 1:**
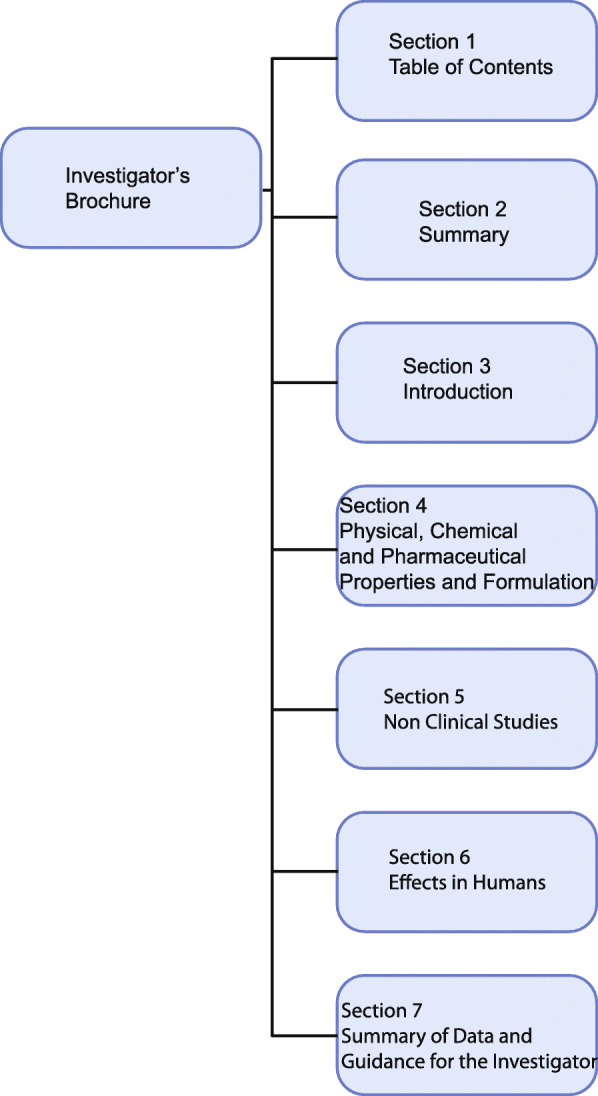
Investigator’s Brochure schematic. Sections 1–7 of the Investigator’s brochure

The purpose of this study is to prepare the required information package for an ICH E6(R2) Investigator’s Brochure (IB), which is part of a clinical trial application (CTA) or an investigational new drug application (IND) for image-guided surgery with IRDye800CW-nimotuzumab. This clinical trial is currently ongoing (NCT04459065). The IB is composed of the following seven sections (Fig. 1): (i) Table of Contents, (ii) Summary, (iii) Introduction, (iv) Physical, Chemical, and Pharmaceutical Properties and Formulation, (v) Nonclinical Studies, (vi) Effects in Humans, and (vii) Summary of Data and Guidance for the Investigator. Data required for Section 4: Physical, Chemical, and Pharmaceutical Properties and Formulation, Section 5: Nonclinical Studies, and Section 6: Effects in Humans are discussed.

The investigational product for this study was IRDye800CW-nimotuzumab, which was developed as an imaging agent for use in image-guided surgery. IRDye800CW-nimotuzumab was constructed by conjugating the near-infrared fluorescent molecule IRDye800CW-NHS ester to the monoclonal antibody nimotuzumab to produce the active pharmacological ingredient IRDye800CW-nimotuzumab. IRDye800CW has an absorbance maximum at 774 nm and emission maximum at 789 nm in PBS, which acts as a fluorescent contrast agent that specifically accumulates on EGFR positive tumors when conjugated to an EGFR specific probe. IRDye800CW-nimotuzumab targets cancer cells, which overexpress EGFR, allowing EGFR positive tumors to be visualized during surgery. EGFR is a cell surface glycoprotein that regulates cell proliferation, survival, and differentiation [1]. Activation of EGFR normally leads to cellular growth, however aberrant activation caused by receptor overexpression, autocrine signalling, or mutation can lead to the development of tumors [1]. EGFR is overexpressed in a number of cancers, particularly in tissue of epithelial origin, including glioma, head and neck, lung, breast, renal, bladder, and prostate cancer [2].

Image-guided surgery is a technology that was developed to improve tumor resection [3]. Currently tumor resection relies on images taken before surgery by computed tomography (CT), positron emission tomography (PET), or magnetic resonance imaging (MRI) [4]. It can be difficult to define tumor margins using static images collected prior to surgery using CT, PET, and/or MRI [4]. Defining tumors using white light illumination and visual inspection can lead to inadequate resection, tumor re-occurrence, and additional surgery(s) [4]. IRDye800CW-nimotuzumab specifically targets EGFR-positive tumors, allowing them to be distinguished from normal surrounding tissue in real-time using near-infrared (NIR) fluorescent imaging. This results in decreased damage to healthy tissue and operation time. In addition, after resection the wound bed can be imaged to determine if there is residual fluorescent malignant tissue, which can then be removed immediately as opposed to waiting for histopathological analysis and a potential second surgery. Targeted imaging agents are designed to specifically recognize molecules expressed on cancer cells like receptors, leading to higher tumor to background ratios.

IRDye800CW-nimotuzumab shares structural similarities to other known IRDye800CW-conjugated probes in clinical trials (Additional File 1), including IRDye800CW-bevacizumab, IRDye800CW-panitumumab, and IRDye800CW-cetuximab. The anti-EGFR antibodies panitumumab and cetuximab are labeled with IRDye800CW and used successfully in image-guided surgery clinical trials to identify EGFR-positive tissues [5, 6]. These EGFR imaging probes show minimal adverse events when patients receive a loading dose of the unlabeled antibody prior to the injection of the imaging probe [7].

Nimotuzumab is marketed and has been administered to over 38,000 patients with limited adverse events reported [8]. Compared to other anti-EGFR antibodies (cetuximab and panitumumab), nimotuzumab has a superior safety profile, particularly with regard to serious cutaneous toxicities [9]. For example, ~ 80% of participants from four different cetuximab studies developed a skin rash that is associated with cetuximab with some grade 3 skin toxicities reported [9]. Panitumumab therapy resulted in 68, 95, 87, and 100% of patients developing skin rash, depending on the dose [9]. In another study, 95% of patients develop skin toxicities, with some grade 3 skin toxicities reported [9]. In comparison, one clinical trial with nimotuzumab reported that 6% of patients developed grade 1 or 2 level skin rash with no grade 3 or 4 level skin toxicities [9]. Further, IRDye 800CW is non-toxic [10].

In this study, we present the formulation and stability of IRDye800CW-nimotuzumab required for Section 4 of the IB and the preclinical safety and pharmacokinetics for Section 5 of the IB. Since IRDye800CW-nimotuzumab has not been injected into humans before to address Section 6: Effects in Humans, we performed a meta-analysis of clinical trials on other IRDye800CW-conjugated antibodies, summarizing indications tested, geographical locations where clinical trials were performed, and adverse events reported.

## Methods

### Reagents

Nimotuzumab was provided by the Center of Molecular Immunology (CIM) (Havana, Cuba). IRDye® 800CW-NHS ester was supplied by LI-COR Biosciences (Lincoln, NE).

### Conjugation of IRDye800CW to nimotuzumab

Four batches of nimotuzumab, each containing five milligrams, were labeled with IRDye® 800CW NHS Ester at a 1:3 (nimotuzumab: IRDye800CW) molar ratio in 1X PBS, pH 7.0, with rotation at room temperature for one hour. Unincorporated IRDye800CW was removed using a Zeba spin desalting column 7 K MWCO (ThermoFisher Scientific). IRDye800CW-nimotuzumab solution was sterilized using a 0.22 μm Ultrafree centrifugal filter (EMD Millipore). The concentration and labeling efficiency were calculated as previously reported [11]. IRDye800CW-nimotuzumab was aliquoted and stored at − 80 °C. Batches 1–3 were used for stability testing and batch 4 was used for toxicity studies. Batch 4 was diluted in a final volume of 200 μL of 0.9% saline prior to injection.

### Purity and molecular weight analysis of IRDye800CW-nimotuzumab

The purity and molecular weight of labeled and unlabeled nimotuzumab were analyzed using microcapillary electrophoresis with an Agilent 2100 Bioanalyzer (Agilent Technologies, Santa Clara, CA), according to the manufacturer’s instructions.

### Stability of IRDye800CW-nimotuzumab

Batches 1–3 of IRDye800CW-nimotuzumab were stored at − 80 °C and 4 °C. Stability of the IRDye800CW-nimotuzumab was tested over time for size and purity using microcapillary electrophoresis (Bioanalyzer), according to the manufacturer’s instructions. Stability was determined as described in Stability testing of new drug substances and products ICH Q1A(R2) [12], where the 95% confidence interval intersects with the acceptance criteria. The pH was determined using a Thermo Scientific™ Orion™ Star A211 pH Benchtop Meter. The pH meter was calibrated at each use. Fluorescence purity was determined using SDS-PAGE where 5 μL of sample diluted 2000-fold in PBS was separated on a 4–20% Mini-PROTEAN® TGX™ Precast Protein Gels for 200 V for 25 min. Volume was determined at the time of dispensing using a scale. Sterility was validated and measured by Keystone Labs Inc. and endotoxin was measured by the Fedoruk Center. Flow cytometry was performed on A-431 with unlabeled and four labeled IRDye800CW-nimotuzuamb batches as previously described [11].

### Mouse xenograft models and in vivo imaging

All mice used in this study were commercially obtained from Charles River Canada and housed in accordance with the University Animal Care Committee (UACC) guidelines (protocol # 20150048). All experiments and euthanasia were performed in accordance with UACC guidelines. All animals were acclimatized for 1 week prior to use. Mice used in all experiments except for toxicity were euthanized using carbon dioxide followed by cervical dislocation. For toxicity mice were anaesthetized with 3% isoflurane and euthanized by exsanguination via cardiac puncture to collect blood for hematology and clinical chemistry. Mice were anesthetized with 3% isoflurane for xenograft implantation and imaging experiments. CD-1 nude female mice were used for fluorescent imaging. For imaging experiments, HT29, MDA-MB-468, and MDA-MB-435 xenografts were prepared by injecting 10^7^ cells suspended in 50 μL of serum free media and mixed with 50 μL of Matrigel basement matrix (Corning) into the right hind flank of 4–6 week old female CD-1 nude mice. Xenografts were monitored with external calipers until they reached 150–300 mm^3^. 0.5 nmoles of IRDye800CW-nimotuzumab was intravenously injected in the tail vein of mice bearing HT29, MDA-MB-468 or MDA-MB-435 xenografts. Mice were imaged 72 and 168 h post injection on a Pearl small animal imager (LI-COR). The EGFR positive xenografts (HT29 and MDA-MB-468) were compared to the negative control xenograft (MDA-MB-435). Each experimental unit had at least three mice (*n* = 3) that were imaged for each xenograft for a total of at least nine mice used for the imaging experiments to get statistically relevant values. No animals and no images were excluded. Mice were randomised based on tumor size to ensure all mice had the same size tumors. There was no blinding in the imaging experiment and confounders were controlled by imaging the animals in the same order. Health and behaviour of the mice were analyzed daily.

### Pharmacokinetics

Twelve 4–6 week old female Balb/c mice were injected intravenously via the tail vein with 0.5 nmole (75 μg) of IRDye800CW-nimotuzumab. Blood was collected from four mice (*n* = 4) after 10 min, 1, 3, 6, 24, 48, 72, 96, 168, and 240 h, and four mice (n = 4) after 5, 15, 30, and 45 min. Four mice (n = 4) were used as a negative control to determine pharmacokinetics. Blood samples were collected from the tail vein in a heparinized capillary tube and the fluorescence was measured using the LI-COR Odyssey scanner. The concentration of IRDye800CW-nimotuzumab was determined using a series of standards in heparinized capillary tubes to generate a calibration curve. No prior criteria were determined to exclude animals and no data collected was excluded. No randomization or blinding was used, and mice were bled in the same order for data point collection to reduce confounders. Mice were analyzed for health and behavior daily. Non-compartmental and bi-exponential analysis was performed using the R [13] package PK [14]. Bi-exponential and non-compartmental models with i.v. bolus input was used to fit pharmacokinetic parameters including area under the curve (AUC), volume of distribution at steady-state (Vss), clearance (CL), and half-life.

### Single dose toxicity study

A single dose of 2 nmoles of IRDye800CW-nimotuzumab was injected via the tail vein of 7–8 week old BALB/c mice to determine acute (day 2) and delayed (day 14) toxicity. All animals in this study were observed regularly for signs of mortality, morbidity, injury, and intake of food and water. Mice were monitored for weight loss and daily clinical observation. Individual body weights were determined and recorded 5 days a week during quarantine and the study period. It was decided prior to the study that animals that started to lose weight before injection would not be included in the study, however no animals were excluded. Forty-eight mice (24 male + 24 female) were divided into five groups (Table 1). Eight mice were used as a baseline control. Four of these mice were sacrificed for acute toxicity baseline at day 2 and four mice for delayed toxicity baseline at day 14. Twenty mice (control group) were treated with saline as the vehicle control. Ten of these mice were sacrificed as controls for acute toxicity at day 2 and ten mice were sacrificed as controls for delayed toxicity at day 14. Twenty mice (treatment group) were injected with 2 nmoles (300 μg) of IRDye800CW-nimotuzumab (batch 4). Ten of these mice were sacrificed for acute toxicity analysis on day 2 and ten mice were sacrificed for delayed toxicity analysis on day 14. There was no randomization or blinding in this study and all animals were collected in the order they were injected within the two time points to minimize confounders.

**Table 1:** Single dose acute and delayed study design

Group	Mice/group	Sex	Compound	Collected^a^ at day 2	Collected^a^ at day 14
Untreated control	4	F	None	2	2
Untreated control	4	M	None	2	2
Vehicle Control	10	F	Saline	5	5
Vehicle control	10	M	Saline	5	5
2 nmoles IRDye800CW-nimotuzumab	10	F	15 mg/kg IRDye800CW-nimotuzumab	5	5
2 nmoles IRDye800CW-nimotuzumab	10	M	15 mg/kg IRDye800CW-nimotuzumab	5	5

^a^number of mice

Organs/tissues (kidneys, spleen, liver, bone, heart, lungs, brain, skin, muscle, and testes/uterus) were inspected, collected, and stored in 10% neutral buffered formalin. Mouse identification was retained with tissues taken during necropsy. The liver, spleen, and kidney were weighed and processed on slides for histopathology evaluation. Samples of each tissue were embedded in paraffin blocks and representative 7 μm sections were mounted on glass microscope slides, stained with hematoxylin and eosin, and the histopathology reviewed by a board-certified pathologist.

Blood was collected while mice were anesthetized with 3% isoflurane prior to euthanasia by exsanguination via cardiac puncture for hematology and clinical chemistry. Blood (50 μL) was collected in a K2 EDTA coated tube for hematology. K2 EDTA coated tubes were made by adding 2.5 μL of a 50 mM stock solution of K2 EDTA. The tubes were dried in a biosafety cabinet overnight. A complete blood count was done on the whole blood samples. A minimum of 500 μL of blood was collected for clinical chemistry analysis into microvette 500 LH heparin-coated tubes (Sarstedt AG & Co). Blood was inverted 8 times and centrifuged at 1500 x g for 10 min. Plasma was collected and sent to Prairie Diagnostic Services (Saskatoon, SK) for clinical chemistry analysis.

Analyses of the clinical chemistry and hematology parameters for differences in means were performed using a non-parametric Kruskal-Wallis test. If a statistically significant difference was present (*p* < 0.05) then comparisons between the vehicle control and treatment groups and baseline and treatment groups were performed using the Dunn’s test. Differences were considered statistically significant if p < 0.05 between the treatment group and both vehicle and baseline groups. Statistical analysis was performed using R project for statistical computing.

### Meta-analysis of clinical trial data of IRDye800CW

A search on pubmed was performed as described in Additional File 2, using search terms “800CW” or “IRDye 800CW” with the clinical trials filter applied. Additional searches for principal investigators involved in clinical trials combined with the search term IRDye800CW resulted in 51 publications. These publications were manually curated for those discussing clinical trials, resulting 17 papers 8 of which contained results on adverse events (Additional File 2).

## Results

### Physical, chemical, and pharmaceutical properties and formulation

**Fig. 2:**
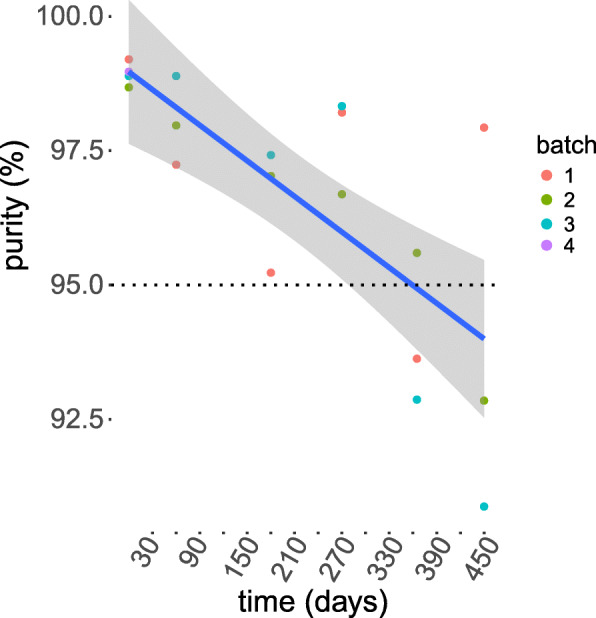
Long term stability of IRDye800CW-nimotuzumab. Four batches of IRDye800CW-nimotzumab were stored at − 80 °C and the stability was analyzed by microcapillary electrophoresis to assess purity over time. The line is the best fit and the shaded area is the 95% confidence interval

The drug substance IRDye800CW-nimotuzumab was made by conjugating the NHS ester IRDye800CW to nimotuzumab. The drug product is formulated in the excipient 0.9% saline for injection. We prepared four different batches of IRDye800CW-nimotzumab. The average labeling ratio of IRDye800CW to nimotuzumab was 1.29 ± 0.03. Protein impurities were assessed by microcapillary electrophoresis. The average purity was 98.9 ± 0.2%. One batch was used for toxicity studies and the other three batches were used to determine stability (Fig. 2). Batches were stable with purities of > 95% for 270 days (9 months) when stored at − 80 °C and one week when stored at 4 °C.

**Fig. 3:**
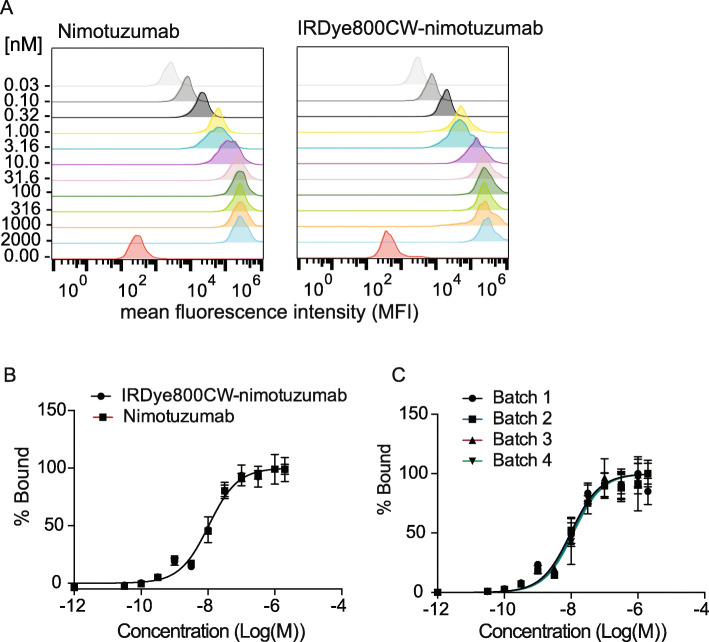
Binding studies of IRDye800CW-nimotuzumab with A-431 cells. Unlabeled nimotuzumab and four batches of IRDye800CW-nimotuzumab were tested for binding to the EGFR positive cell line A-431 using flow cytometry to test reproducibility of batch formulation. **a** and **b** show IRDye800CW-nimotuzumab binding compared to unlabeled nimotuzumab. **c** Batch-to-batch variability of IRDye800CW-nimotuzumab in cell binding. The error bars represent the standard deviation

To assess whether the conjugate IRDye800CW-nimotuzumab was functional, samples were tested for binding to the EGFR positive cell line A-431 using flow cytometry. All four batches of IRDye800CW-nimtotuzumab bound to the A-431 cell line with the following affinities: Batch 1 = 9 ± 2 nM, Batch 2 = 10 ± 1 nM, Batch 3 = 12 ± 3 nM, and Batch 4 = 12 ± 2 nM (Fig. 3). There were no significant differences (*p* > 0.05) between K_D_ values of batches. Labeling nimotuzumab with IRDye800CW did not have a significant affect (p > 0.05) on its ability to bind to A-431 when compared to the unlabeled antibody (K_D_ of 10.5 ± 0.7 nM).

**Fig. 4:**
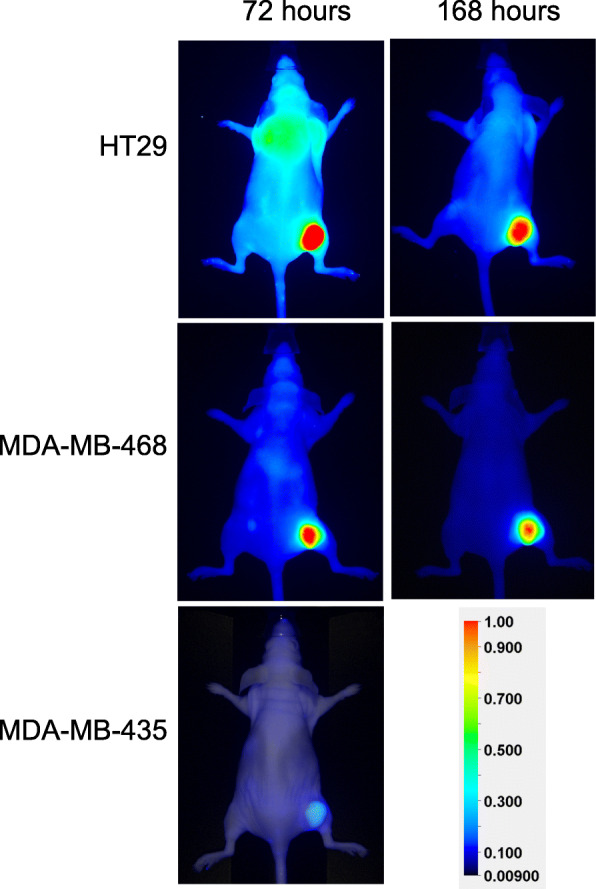
IRDye800CW-nimotuzumab imaging in mice bearing HT29, MDA-MB-468 and MDA-MB-435 xenografts. IRDye800CW-nimotuzumab was injected into CD-1 nude mice bearing EGFR positive xenografts **a**) HT-29, **b**) MDA-MB-468, and an EGFR negative xenograft **c**) MDA-MB-435 and imaged at 72 and 168 h post injection. Scale represents the mean fluorescence intensity

Nimotuzumab-IRDye800CW accumulated in EGFR positive xenografts generated from HT29 and MDA-MB-468 cell lines and not in the EGFR- negative xenograft generated from MDA-MB-435 (Fig. 4). We previously showed that MDA-MB-435 has very low EGFR expression and the levels of EGFR expression are similar in HT29 and MDA-MB-468 cell lines [11].

The batches of IRDye800CW-nimotuzumab were reproducibly prepared at high purity. There was no significant difference in the affinities to EGFR positive cells between any of the batches produced or between the batches and unlabeled nimotuzumab, indicating the robustness of the formulation process. The probe was stable for nine months when stored at − 80 °C and for one week when stored at 4 °C, which gives a starting point for determining the shelf-life. This information was used to define specifications (Table 2).

**Table 2:** Specifications for IRDye800CW-nimotuzumab

Test parameter	Method	Acceptance Limits
Visual appearance	Visual inspection	Clear, green solution, free from visible particulates
pH	pH meter	6.5–7.5
Labelling ratio (Dye:Protein)	UV	0.7–2.0
Strength (mg/mL)	UV	10 (±20%)
Volume (mL)	Weight	5 (±10%)
Functional assay (Binding to cells)	Flow cytometry	K_D,sample_/K_D,ref_ × 100% < = 200% > = 50%
Identity and purity (protein)	Micro-capillary electrophoresis	150 kDa (±10%) Peak > = 95%
Purity (fluorescence)	SDS-PAGE	> = 90%
Bacterial endotoxin	USP < 85>	< 16 EU/mL
Sterility	USP < 71>	No growth

### Nonclinical studies: pharmacokinetics of IRDye800CW-nimotuzumab

**Fig. 5:**
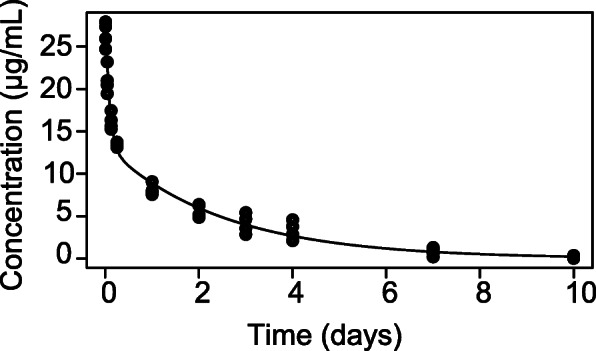
Pharmacokinetic analysis of IRDye800CW-nimotuzumab in Balb/c mice. IRDye800CW-nimotuzumab was injected into BALB/c mice and blood was collected over time. The concentration of IRDye800CW-nimotuzumab in the blood was measured and plotted vs time. The data was fit using bi-exponential analysis

Pharmacokinetics of IRDye800CW-nimotuzumab was studied in normal BALB/c mice. Twelve mice were injected with 0.5 nmoles (75 μg) of IRDye800CW-nimotuzumab. Blood was collected over time and the mean fluorescence was measured and analyzed using bi-exponential and non-compartmental analyses. IRDye800CW-nimotuzuamb has a non-compartmental half-life of 38 ± 1.5 h. Area under the curve (AUC) was 36 ± 2 μg•day/mL and AUC to infinity was 35.7 μg•day/mL. Clearance (CL) was 2 ± 1 mL/day and volume of distribution at steady state (Vss) was 4.8 ± 0.2 mL. The pharmacokinetics of IRDye800CW-nimotuzumab was also analyzed by a bi-exponential analysis (Fig. 5), which gave a t_1/2_ alpha of 1.5 h and t_1/2_ beta of 40.8 h. The AUC was 34.5 μg•day/mL. The systemic clearance (CL) was 2.2 mL/day and the volume distribution at steady state (Vss) was calculated to be 5.3 mL.

### Nonclinical studies: acute and delayed toxicity of IRDye800CW-nimotuzumab

Single does acute (2-day) and delayed (14-day) toxicity of IRDye800CW-nimotuzumab were studied in normal BALB/c mice. Groups of mice were treated as shown in Table 1. Saline was injected as the vehicle control and untreated mice were used as a baseline. Mice were monitored and weighed daily one week before and for the duration of the study (Additional File 3). No significant weight gain or loss was observed between the IRDye800CW-nimotuzumab and vehicle or baseline groups during the study. All animals survived to the scheduled necropsy. There was no apparent anemia, weight loss, agitation, tachypnea, GI disturbances, or neurological dysfunction in any of the mice groups. All mice looked healthy and displayed normal behavior throughout. Tissues were collected for histopathology and blood was collected for CBC and clinical chemistry.

Major organs (liver, spleen, kidney, heart, brain, lungs, muscle, skin, bone, and uterus/testis) were collected. The liver, spleen, and kidney were weighed (Additional File 4) and processed for histopathological analysis. There was no significant difference in organ weight between treatment groups (*p* > 0.05). Histopathological examination of necropsy stained slices showed no damage to organs following administration of 2 nmoles of IRDye800CW-nimotuzumab (a 25-fold mass excess of the projected human dose). Single dose acute (2 Day) and delayed (14 Day) toxicity analysis showed that IRDye800CW-nimtuzumab was non-toxic in normal BALB/c mice.

To assess the clinical chemistry for toxicity of IRDye800CW-nimotuzumab, blood was collected via cardiac puncture and the plasma was separated and analyzed. At both 2 days and 14 days post injection there were no differences in the means (p > 0.05) of any of the blood chemistry parameters in females or males when the treatment groups were compared to the baseline and vehicle group. These results are summarized in Tables 3, Table 4 and Additional File 5.

**Table 3:** Clinical chemistry results in male BALB/c mice injected with IRDye800CW-nimotuzumab

Parameter	baseline	day 2		day 14		(male)
		vehicle	Nz-800CW	vehicle	Nz-800CW	
	mean ± sd	mean ± sd		mean ± sd		unit
A:G Ratio	1.5 ± 0.1	1.6 ± 0.1	1.6 ± 0.2	1.6 ± 0.0	1.7 ± 0.1	
ALT	24.8 ± 5.0	27.6 ± 4.0	25.2 ± 4.1	27.2 ± 3.3	22.8 ± 2.6	U/L
Albumin	28.5 ± 0.6	29.4 ± 1.1	29.4 ± 1.5	30.8 ± 1.8	31.5 ± 0.6	g/L
Alk Phos	114 ± 9	131 ± 4	122 ± 11	123 ± 3	114 ± 7	U/L
Amylase	2893 ± 836	3167 ± 247	2883 ± 127	3130 ± 223	3692 ± 764	U/L
CK	310 ± 127	255 ± 187	217 ± 78	380 ± 131	356 ± 154	U/L
Calcium	2.4 ± 0.3	2.5 ± 0.1	2.5 ± 0.1	2.6 ± 0.1	2.5 ± 0.1	mmol/L
Chloride	105 ± 1	105 ± 2	105 ± 0.8	102 ± 1.5	104 ± 1.7	mmol/L
Cholesterol	3.3 ± 0.2	3.3 ± 0.2	3.4 ± 0.1	3.2 ± 0.2	3.2 ± 0.2	mmol/L
Creatinine	15.8 ± 3.0	15.5 ± 2.6	17.2 ± 2.4	17.8 ± 2.3	13.4 ± 2.3	μmol/L
GLDH	7.2 ± 1.3	9.8 ± 1.9	8.6 ± 1.1	7.0 ± 0.7	6.0 ± 0.8	U/L
Globulin	18.8 ± 1.0	19.0 ± 1.2	19.0 ± 1.4	19.2 ± 1.1	18.8 ± 1.0	g/L
Glucose	15.4 ± 2.7	12.8 ± 1.7	13.8 ± 1.6	12.8 ± 2.8	13.8 ± 1.1	mmol/L
Lipase	27.0 ± 0.8	27.4 ± 2.5	25.0 ± 4.2	28.4 ± 2.3	28.8 ± 1.8	U/L
Magnesium	1.1 ± 0.1	1.1 ± 0.1	1.1 ± 0.1	1.2 ± 0.1	1.1 ± 0.1	mmol/L
Na:K Ratio	35.5 ± 1.3	37.8 ± 2.8	41.5 ± 3.7	40.6 ± 7.1	38.5 ± 1.9	
Phosphorus	2.9 ± 0.3	2.3 ± 0.5	2.5 ± 0.3	3.0 ± 0.2	2.6 ± 0.3	mmol/L
Potassium	4.2 ± 0.2	4.1 ± 0.3	3.6 ± 0.3	3.7 ± 0.7	4.0 ± 0.2	mmol/L
Sodium	151 ± 2	154 ± 3	150 ± 2	149 ± 2	151 ± 2	mmol/L
Total Bilirubin	0.1 ± 0.2	0.3 ± 0.4	0.7 ± 0.4	0.2 ± 0.2	0.4 ± 0.5	μmol/L
Total Protein	47.2 ± 1.0	48.4 ± 0.6	48.4 ± 0.6	50.0 ± 2.8	49.8 ± 1.6	g/L
Urea	8.7 ± 0.2	10.4 ± 0.6	9.6 ± 1.1	9.8 ± 1.5	9.0 ± 1.5	mmol/L

*Abbreviations*: *Nz-800* IRDye800CW-nimotuzumab, *A:G* albumin to globulin, *ALT* alanine aminotransferase, *alk phos* alkaline phosphatase, *CK* creatin kinase, *GLDH* glutamate dehydrogenase, *Na:K* sodium to potassium

**Table 4:** Clinical chemistry results in female BALB/c mice injected with IRDye800CW-nimotuzumab

Parameter	baseline	day 2		day 14		(female)
		vehicle	Nz-800CW	vehicle	Nz-800CW	
	mean ± sd	mean ± sd		mean ± sd		unit
A:G Ratio	2.0 ± 0.1	1.9 ± 0.4	2.1 ± 0.3	2.4 ± 0.2	2.3 ± 0.2	
ALT	27.2 ± 4.8	41.5 ± 21.1	26.2 ± 2.9	23.8 ± 7.2	30.5 ± 11.2	U/L
Albumin	32.0 ± 2.2	31.8 ± 3.4	32.2 ± 1.5	32.0 ± 0.7	33.5 ± 0.6	g/L
Alk Phos	123 ± 15	150 ± 20	164 ± 12	128 ± 8	134 ± 10	U/L
Amylase	2332 ± 292	2537 ± 139	2819 ± 480	2335 ± 140	2307 ± 123	U/L
CK	413 ± 182	1248 ± 391	696 ± 254	805 ± 661	352 ± 193	U/L
Calcium	2.4 ± 0.2	2.4 ± 0.1	2.1 ± 0.3	2.5 ± 0.1	2.3 ± 0.1	mmol/L
Chloride	105 ± 0.6	101 ± 6	108 ± 5	106 ± 2	106 ± 1	mmol/L
Cholesterol	2.5 ± 0.2	2.3 ± 0.3	2.2 ± 0.1	2.3 ± 0.2	2.4 ± 0.2	mmol/L
Creatinine	17.8 ± 3.0	15.8 ± 1.7	13.5 ± 1.7	15.0 ± 3.3	16.2 ± 1.3	μmol/L
GLDH	10.0 ± 2.9	13.5 ± 4.0	13.0 ± 1.7	9.0 ± 2.8	8.2 ± 3.0	U/L
Globulin	15.8 ± 0.5	16.5 ± 1.7	15.5 ± 1.7	13.6 ± 1.3	14.5 ± 1.3	g/L
Glucose	15.2 ± 1.6	13.4 ± 1.6	12.9 ± 1.5	15.0 ± 1.1	14.8 ± 0.6	mmol/L
Lipase	25.8 ± 3.6	26.8 ± 2.9	26.0 ± 2.2	27.0 ± 2.5	26.8 ± 2.2	U/L
Magnesium	1.2 ± 0.1	1.1 ± 0.1	1.0 ± 0.1	1.1 ± 0.1	1.2 ± 0.1	mmol/L
Na:K Ratio	39.8 ± 1.3	33.5 ± 5.8	41.0 ± 7.1	42.4 ± 4.6	42.3 ± 3.8	
Phosphorus	3.3 ± 0.3	2.7 ± 0.4	2.3 ± 0.3	2.8 ± 0.4	2.8 ± 0.3	mmol/L
Potassium	3.7 ± 0.1	4.3 ± 0.7	3.7 ± 0.6	3.6 ± 0.3	3.6 ± 0.4	mmol/L
Sodium	149 ± 0.5	144 ± 5	149 ± 4	150 ± 2	152 ± 1.5	mmol/L
Total Bilirubin	0.4 ± 0.2	0.0 ± 0.0	0.3 ± 0.5	0.2 ± 0.3	0.6 ± 0.8	μmol/L
Total Protein	47.8 ± 2.2	48.2 ± 1.7	47.8 ± 1.3	45.6 ± 1.9	48.0 ± 1.4	g/L
Urea	9.7 ± 0.9	10.9 ± 1.4	10.8 ± 1.0	10.2 ± 0.7	9.0 ± 1.0	mmol/L

*Abbreviations*: *Nz-800* IRDye800CW-nimotuzumab, *A:G* albumin to globulin, *ALT* alanine aminotransferase, *alk phos* alkaline phosphatase, *CK* creatin kinase, *GLDH* glutamate dehydrogenase, *Na:K* sodium to potassium

To assess hematology toxicity of IRDye800CW-nimotuzumab, blood was collected after injection via cardiac puncture on day 2 or day 14. Whole blood was tested and analyzed for complete blood counts (CBC). Hematological results are presented in Table 5, Table 6, and Additional File 6. A significant increase (IRDye800-nimotuzumab = 2.2 ± 0.5, vehicle = 1.1 ± 0.3, baseline = 0.9 ± 0.4 × 10^9^/L; *p* < 0.01) was seen in the white blood cell count between IRDye800CW-nimtozumab and the control and vehicle at 2 days in female mice. This difference was not present at 14-days and was not present in male mice at 2-day or 14-days. There was no difference (*p* > 0.05) in any of the other hematology parameters.

**Table 5:** Hematology analyses from male BALB/c mice injected with IRDye800CW-nimotuzumab

Parameter	baseline	day 2		day 14		(male)
		vehicle	Nz-800CW	vehicle	Nz-800CW	
	mean ± sd	mean ± sd		mean ± sd		unit
GR	5.5 ± 0.9	2.7 ± 1.0	4.3 ± 1.4	2.7 ± 0.7	5.4 ± 1.6	%
Hct	0.5 ± 0.1	0.5 ± 0.1	0.5 ± 0.1	0.5 ± 0.0	0.5 ± 0.0	L/L
Hgb	156 ± 19	146 ± 20	159 ± 23	166 ± 4	169 ± 3	g/L
LY	80.3 ± 2.8	85.2 ± 4.5	81.5 ± 3.2	87.5 ± 1.6	80.6 ± 3.6	%
MCH	18.7 ± 0.2	19.1 ± 0.3	19.0 ± 0.2	18.9 ± 0.1	18.6 ± 0.2	gp
MCHC	321 ± 1	321 ± 1	322 ± 1	322 ± 0.1	322 ± 0.1	g/L
MCV	58.2 ± 0.6	59.4 ± 0.8	59.0 ± 0.7	58.8 ± 0.2	57.6 ± 0.6	fL
MO	14.2 ± 3 .0	12.1 ± 3.8	14.1 ± 2.0	9.8 ± 1.7	13.9 ± 2.6	%
Plt	860 ± 228	900 ± 164	809 ± 219	923 ± 90	1001 ± 78	× 10^9^/L
RBC	8.8 ± 0.2	8.9 ± 0.4	8.7 ± 0.3	8.7 ± 0.1	9.1 ± 0.2	× 10^12^/L
RDW	15.6 ± 0.3	15.9 ± 0.8	15.9 ± 0.6	16.9 ± 1.9	19.6 ± 5.6	%
WBC	1.4 ± 0.3	2.1 ± 0.7	1.5 ± 0.8	1.6 ± 1.0	2.5 ± 1.1	×10^9^/L

*Abbreviations*: *Nz-800* IRDye800CW-nimotuzumab, *GR* granulocytes, *Hct* hematocrits, *hgb* hemoglobin, *LY* lymphocytes, *MCH* mean corpuscular hemoglobin, *MCHC* mean corpuscular hemoglobin concentration, *MCV* mean corpuscular volume, *MO* monocytes, *Plt* platelets, *RBC* red blood cells, *RDW* red blood cell distribution width, *WBC* white blood cell

**Table 6:** Hematology analyses from female BALB/c mice injected with IRDye800CW-nimotuzumab

Parameter	baseline	day 2		day 14		(female)
		vehicle	Nz-800CW	vehicle	Nz-800CW	
	mean ± sd	mean ± sd		mean ± sd		unit
GR	7.0 ± NA	4.6 ± 1.4	4.1 ± 2.3	2.8 ± 0.3	3.1 ± 1.6	%
Hct	0.5 ± 0.1	0.4 ± 0.1	0.4 ± 0.0	0.5 ± 0.1	0.5 ± 0.0	L/L
Hgb	160 ± 22	138 ± 22	140 ± 12	156 ± 19	161 ± 13	g/L
LY	77.8 ± NA	79.8 ± 4.3	82.9 ± 2.3	85.2 ± 1.2	83.6 ± 6.9	%
MCH	18.9 ± 0.3	18.6 ± 1.2	17.2 ± 0.3	19.0 ± 0.5	19.2 ± 0.2	gp
MCHC	322 ± 1.3	320 ± 1.7	321 ± 0.6	321 ± 1.2	322 ± 0.5	g/L
MCV	58.7 ± 1.0	58.0 ± 3.5	53.7 ± 1.0	59.1 ± 1.6	59.5 ± 0.6	fL
MO	15.2 ± NA	15.5 ± 2.9	13.0 ± 1.2	12.1 ± 1.2	13.4 ± 5.3	%
Plt	703 ± 173	602 ± 160	453 ± 318	630 ± 217	711 ± 137	×10^9/L
RBC	9.0 ± 0.4	8.2 ± 0.5	7.5 ± 0.8	8.8 ± 0.2	8.8 ± 0.6	×10^12/L
RDW	19.3 ± 7.1	19.1 ± 1.2	20.8 ± 0.9	15.7 ± 0.7	15.5 ± 0.3	%
WBC	0.9 ± 0.4	1.1 ± 0.3	2.2 ± 0.5	1.9 ± 1.5	1.0 ± 0.4	× 10^9/L

*Abbreviations*: *Nz-800* IRDye800CW-nimotuzumab, *GR* granulocytes, *Hct* hematocrits, *hgb* hemoglobin, *LY* lymphocytes, *MCH* mean corpuscular hemoglobin, *MCHC* mean corpuscular hemoglobin concentration, *MCV* mean corpuscular volume, *MO* monocytes, *Plt* platelets, *RBC* red blood cells, *RDW* red blood cell distribution width, *WBC* white blood cell

### Effects in humans: meta-analysis of clinical trials with IRDye800CW

We showed that IRDye800CW-nimotuzmab was safe in BALB/c mice, however it has never been injected into humans. Furthermore, IRDye800CW in its carboxylate form is safe up to a concentration of 20 mg/kg when injected into rats (> 500-fold higher than used in this study) [10]. Also, since there have been numerous other IRDye800CW probes used in clinical trials (Additional File 1), we performed meta-analysis of these other IRDye800CW probes in clinical trials to address the likely safety of IRDye800CW-nimotuzumab.

**Fig. 6:**
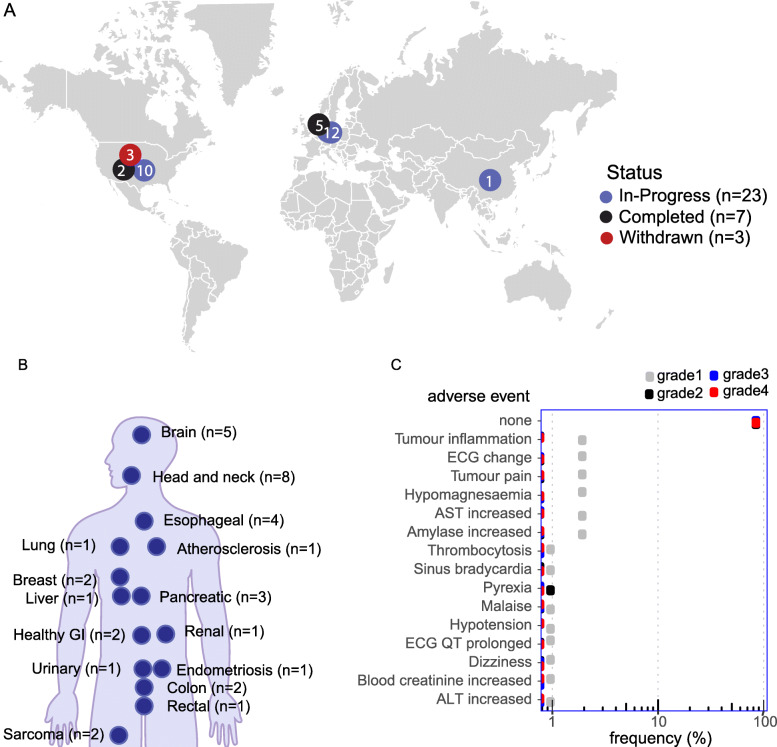
Safety of IRDye800CW. **a** A world map schematic showing the number of in-progress, completed, and withdrawn clinical trial with IRDye800CW. The map was produced using R Studio version 3.3.2 (https://www.r-project.org). **b** Diagram of indications tested in clinical trials with IRDye800CW. **c** Adverse events and grade level associated with IRDye800CW from clinical trial data

A search on clinicaltrials.gov for clinical trials with IRDye800CW resulted in 34 clinical trials (Additional File 7). Twenty-three were in-progress with ten in the USA, twelve in the Netherlands, and one in China, (Fig. 6a). Only three clinical trials have been terminated due to logistics, which involved IRDye800CW-cetuximab (Fig. 6a). The most predominant indication is head and neck cancer (8 clinical trials) followed by brain (5 clinical trials) and esophageal (4 clinical trials) (Fig. 6b and Additional File 8). Of the 34 clinical trials identified, six are complete with one trial in the USA and five trials in the Netherlands.

These 34 clinical trials involve eight different imaging probes (Additional Files 7 and 9). The majority of trials (*n* = 13) involved IRDye800CW-bevacizumab (5 completed, 8 in-progress), which targets Vascular Endothelial Growth Factor (VEGF). The second most common probe IRDye800CW-pantitumumab targets EGFR and is being evaluated in six clinical trials. A second anti-EGFR probe IRDye800CW-cetuximab is being evaluated in two clinical trials; three clinical trials with this probe have been terminated due to logistics. Of the 34 clinical trials with IRDye800CW, no clinical trial has been withdrawn for safety reasons. Image-guided surgery is an expanding field with more clinical trials in-progress than completed (Fig. 6a and Additional File 9).

To assess the expected safety of IRDye800CW-nimotuzumab, we performed a meta-analysis of adverse events (AEs) in patients treated with other IRDye800CW-conjugated antibodies. The analysis involved 102 patients from eight clinical trials that reported data on adverse events (Fig. 6c, Additional File 2) [15–22]. AEs that were potentially caused by the IRDye800CW-antibody were collected. MedDra version 21 was used to consolidate AEs based on low-level terms to the preferred term. No serious adverse events grade 3+ were reported [15–22]. The majority 88/102 (86%) of patients did not experience any AEs no serious (grade 3+) adverse events were reported. There was a single (1%) grade 2 adverse event, pyrexia (fever). The most frequent grade 1 AEs occurred less than 2% [15–22]. In general, it was found that adverse events were similar to the parent antibody [7].

The parent antibody, nimotuzumab is registered in over 30 non-ICH countries. Post-marketing safety experience with nimotuzumab shows that serious adverse events (SAE) are rare and occur in less than 1% of patients [8].

## Discussion

EGFR is overexpressed in a number of cancers. Many of these cancers are not responsive to therapies due to mutations and/or acquired resistance making surgical resection the only option. During resection of cancerous tissue, it can be difficult to distinguish between diseased and healthy tissue. Near-infrared fluorescence, image-guided surgery can be used to visualize cancerous tissues through specific binding of a fluorescent probe. EGFR is commonly over-expressed in a number of cancers [2]. Panitumumab and cetuximab are EGFR antibodies have been labeled with IRDye800CW and are currently in clinical trials for surgical navigation. Both conjugated antibodies have shown minimal toxicities [7, 13]. Nimotuzumab is an antibody that specifically binds to EGFR. Here, we conjugate IRDye800CW to nimotuzumab to study the pre-clinical properties for use as a fluorescent probe in image-guided surgery.

The elimination half-life of IRDye800CW-nimotuzumab calculated using both non-compartment and two-compartment modeling (38 h and 40.8 h) in mouse was in the range of published results for nimotuzumab at doses of 100–400 mg in humans (34–75 h) [23–25]. This is consistent with previously reported IRDye800CW-labeled EGFR antibodies showing their pharmacokinetic properties were similar to that of the parent antibody [7].

Acute and delayed toxicity of IRDye800CW-nimtozumab was tested in BALB/c mice. There was an increase in the white blood cell count in the female mice at two days. By 14 days this difference was no longer present. These differences were not associated to histopathological findings or clinical observations of significance and therefore were not considered pathologically significant. There were no other differences in the hematology results of the other parameters and treatment groups.

The two most relevant probes to compare IRDye800CW-nimotuzumab with are IRDye800CW-panitiumamab and IRDye800CW-cetuximab as they also bind EGFR. The dose used in the preclinical toxicity studies was 15 mg/kg IRDye800CW-nimotuzumab (1.2 mg/kg or 45 mg/m^2^ human equivalent dose). A dose at the upper range of clinical studies in humans using IRDye800CW-panitumumab (0.06, 0.5, and 1 mg/kg) or IRDye800CW-cetuximab (2.5 mg/m^2^, 25 mg/m^2^ and 62.5 mg/m^2^) [7]. In terms of safety, nimotuzumab when given alone as a therapy, has minimal skin toxicities compared to other EGFR antibodies where skin toxicities occur in the majority of the patients (~ 80% for cetuximab and ~ 68% for panitumumab) and can be quite severe with some grade 3 reactions reported [9]. In studies with IRDye800CW-cetuximab and IRDye800CW-panitumumab, pre-injection with unlabeled antibody is performed prior to injection of the IRDye800CW probe to separate adverse events from the antibody with adverse events from the IRDye800CW-labeled antibody. Two infusion reactions have been observed for IRDye800CW-labeled cetuximab (*n* = 14) and none for IRDye800CW-labeled panitumumab (*n* = 15) [7]. No adverse reactions above grade 1 are observed for either of the IRDye800CW-labeled antibodies [7]. In general, the low toxicity observed for other anti-EGFR antibodies conjugated with IRDye800CW agrees with the toxicity results we observed in mice.

Safety studies with IRDye800CW-cetuximab and IRDye800CW panitumumab show that the parent antibodies are similar to the IRDye800CW labeled antibodies [7], indicating that labeling antibodies with IRDye800CW does not increase their toxicity. In humans, use of the parent antibody, nimotuzumab has been shown in 38,629 patients, only 36 (0.09%) had serious adverse events (SAE) related to the antibody [8]. In an observational prospective post registration clinical trial with 577 patients [8], only four SAE were reported (0.7%). Adverse events (AE) are reported to be mild and occur in 10–15% of patients. The most frequently reported AE of a phase IV clinical trial with 127 patients were headache, increased transaminase, fever and skin rash; all were mild intensity [8]. It is important to note that the post marketing experience is from nimotuzumab administered at a clinical dose for multiple cycles. For image-guided surgery, nimotuzumab will be injected at a subclinical dose once.

## Conclusions: brief summary and potential implications

Here, we show that IRDye800CW-nimotuzumab is a safe molecule with potential to be used in image-guided surgery. Currently approved EGFR antibodies are being used in clinical trials with great success. IRDye800CW-nimotuzumab has a better safety profile than these parent antibodies. This study provides the basis for Sections 4, 5, and 6 of the Investigator’s Brochure for a clinical trial application or investigational new drug application to use IRDye800CW-nimotuzumab in image-guided surgery of EGFR positive tumors.

## Supplementary Information

**Additional file 1.** Clinical trials by probe. A table listing the clinical trials by probe with IRDye800CW and the number (n) of clinical trials.

**Additional file 2.** Schematic of how the meta-analysis on clinical trials with IRDye800CW was done and tables showing the clinical trials with IRD800CW with results published on pubmed.

**Additional file 3.** Mouse weights from toxicity studies. Graphs showing the weights of the mice used for the toxicity experiments prior to and during the experiments.

**Additional file 4.** Weight of mouse liver, kidney, and spleen from toxicity studies. Graphs showing the weights of the liver, spleen and kidney of the mice used for the toxicity experiments.

**Additional file 5.** Clinical chemistry results from the toxicity study. Sodium, potassium, sodium: potassium (Na:K) ratio, chloride, calcium, phosphorus magnesium, urea, creatinine, amylase, lipase, glucose, cholesterol, bilirubin, alkaline phosphatase (Alk Phos), alanine aminotransferase (ALT), glutamate dehydrogenase (GLDH), CK (creatine kinase), protein, albumin, globulin and albumin:globulin (A:G) ratio measurements from the IRDye800CW-nimotuzumab (800CW-Nz) toxicity studies.

**Additional file 6.** Hematology results from toxicity study. White blood cells (WBC), lymphocytes (LY), monocytes (MO), granulocytes (GR), red blood cells (RBC) and red blood cell distribution width (RDW), hematocrits (Hct), platelets (Plt), haemoglobin (Hgb), mean corpuscular haemoglobin (MCH), mean corpuscular haemoglobin count (MCHC) and mean corpuscular volume (MCV) measurements from the IRDye800CW-nimotuzumab (800CW-Nz) toxicity studies.

**Additional file 7.** Table 2: Clinical trials on clinicaltrials.gov accessed 20,190,422 search term: “800CW OR IRDYE800CW OR IRDYE”.

**Additional file 8.** Indications of clinical trials with IRDye800CW. A table listing the clinical trials, indications and the number of indications (n).

**Additional file 9.** Clinical trials by probe and trial status. A table listing clinical trials by probe of probes similar to IRDye800CW-nimotuzumab showing trials ‘completed’, ‘in progress’ and ‘terminated’ or ‘withdrawn’.

## Data Availability

All data generated or analyzed during this study are included in this published article [and its supplementary information files].

## Abbreviations

EGFR: Epidermal growth factor receptor
IB: Investigator’s Brochure
CT: Computed tomography
PET: Positron emission tomography
MRI: Magnetic resonance imaging
NIR: Near infrared
CIM: Center of Molecular Immunology
PBS: Phosphate buffered saline
UACC: University animal care committee
CD-1: Cluster difference 1
EDTA: Ethylenediaminetetraacetic acid
GI: Gastrointestinal intestinal
Nz-800: IRDye800CW-nimotuzumab
t_1/2_: Half-life
A:G: Albumin to globulin
ALT: Alanine aminotransferase
alk phos: Alkaline phosphatase
CK: Creatine kinase
GLDH: Glutamate dehydrogenase
Na:K: Sodium to potassium
CBC: Complete blood counts
GR: Granulocytes
Hct: Hematocrits
hgb: Hemoglobin
LY: Lymphocytes
MCH: Mean corpuscular hemoglobin
MCHC: Mean corpuscular hemoglobin concentration
MCV: Mean corpuscular volume
MO: Monocytes
Plt: Platelets
RBC: Red blood cells
RDW: Red blood cell distribution width
WBC: White blood cell
VEGF: Vascular Endothelial Growth Factor
SAE: Serious adverse events
AE: Adverse events
