# Supplementary material for: Pre-clinical study of IRDye800CW-nimotuzumab formulation, stability, pharmacokinetics, and safety
Source: BMC Cancer. 2021 Mar 12;21:270. doi: 10.1186/s12885-021-08003-3 (PMC7953729; doi:10.1186/s12885-021-08003-3)
Supplement: Supplementary file 1 — Additional file 1. Clinical trials by probe. A table listing the clinical trials by probe with IRDye800CW and the number (n) of clinical trials. [file 12885_2021_8003_MOESM1_ESM.pdf]

Additional File 2

Clinical trials by probe.

parent	conjugate	class	n	nct_id
Bevacizumab*	IRDye800CW	antibody	13	NCT03913806, NCT03620292, NCT03877601, NCT03558724, NCT02583568, NCT02113202, NCT02129933, NCT02743975, NCT01972373, NCT01508572, NCT02975219, NCT03757507
Panitumumab*	IRDye800CW	antibody	6	NCT03582124, NCT03405142, NCT03384238, NCT03510208, NCT03733210, NCT02415881
Cetuximab*	IRDye800CW	antibody	5	NCT03134846, NCT02855086, NCT02736578, NCT01987375, NCT03923881
ABY-029	IRDye800CW	affibody	3	NCT03282461, NCT03154411, NCT02901925
KSP**	IRDye800CW	peptide	3	NCT03643068, NCT03161418, NCT03852576
BBN	IRDye800CW/68Ga	peptide	2	NCT03407781, NCT02910804
Girentuximab	IRDye800CW/Indium-111-DOTA	antibody	1	NCT02497599
Labetuzumab	IRDye800CW/Indium-111-DOTA	antibody	1	NCT03699332

**Structurally similar to IRDye800CW-nimotuzumab*

***KSP indicates one of three probes: KSP-QRH-E3, KSP-910638G, or KSP/QRH dimer*
