# Supplementary material for: Pre-clinical study of IRDye800CW-nimotuzumab formulation, stability, pharmacokinetics, and safety
Source: BMC Cancer. 2021 Mar 12;21:270. doi: 10.1186/s12885-021-08003-3 (PMC7953729; doi:10.1186/s12885-021-08003-3)
Supplement: Supplementary file 2 — Additional file 2. Schematic of how the meta-analysis on clinical trials with IRDye800CW was done and tables showing the clinical trials with IRD800CW with results published on pubmed. [file 12885_2021_8003_MOESM2_ESM.pdf]

Additional File 1

pubmed search term and number of results (n)
total = 51

800CW OR IRDye 800CW AND Clinical Trial filter
n = 7

Nagengast AND (IRDye OR 800CW OR IRDye800CW OR IRDye800)
n = 8

van Dam AND (IRDye OR 800CW OR IRDye800CW OR IRDye800)
n = 12

Peter Mulders AND (IRDye OR 800CW OR IRDye800CW OR IRDye800)
n = 6

Eben Rosenthal AND (IRDye OR 800CW OR IRDye800CW OR IRDye800)
n = 29

Deling Li AND (IRDye OR 800CW OR IRDye800CW OR IRDye800)
n = 1

Eric Henderson AND (IRDye OR 800CW OR IRDye800CW OR IRDye800)
n = 2

David Roberts AND (IRDye OR 800CW OR IRDye800CW OR IRDye800)
n = 2

Witjes AND (IRDye OR 800CW OR IRDye800CW OR IRDye800)
n = 0

Danielle urgeon AND (IRDye OR 800CW OR IRDye800CW OR IRDye800)
n = 0

Joseph Paydarfar AND (IRDye OR 800CW OR IRDye800CW OR IRDye800)
n = 0

Unique and relavent clinical trials with results
n = 17

Pubmed ID
29556334
28119364
27879266
28404198
29623552
29721096
29967260
27499920
26120042
26074273
28446503
27830425
27587708
25904751
29721094
29667116
29721070

Unnique and relavent clinical trials with results that report adverse events
n = 8

Pubmed ID
29623552
29721070
25904751
29967260
29667116
28119364
28404198
29556334

accessed September 14, 2018
