# Supplementary material for: Pre-clinical study of IRDye800CW-nimotuzumab formulation, stability, pharmacokinetics, and safety
Source: BMC Cancer. 2021 Mar 12;21:270. doi: 10.1186/s12885-021-08003-3 (PMC7953729; doi:10.1186/s12885-021-08003-3)
Supplement: Supplementary file 3 — Additional file 3. Mouse weights from toxicity studies. Graphs showing the weights of the mice used for the toxicity experiments prior to and during the experiments. [file 12885_2021_8003_MOESM3_ESM.pdf]

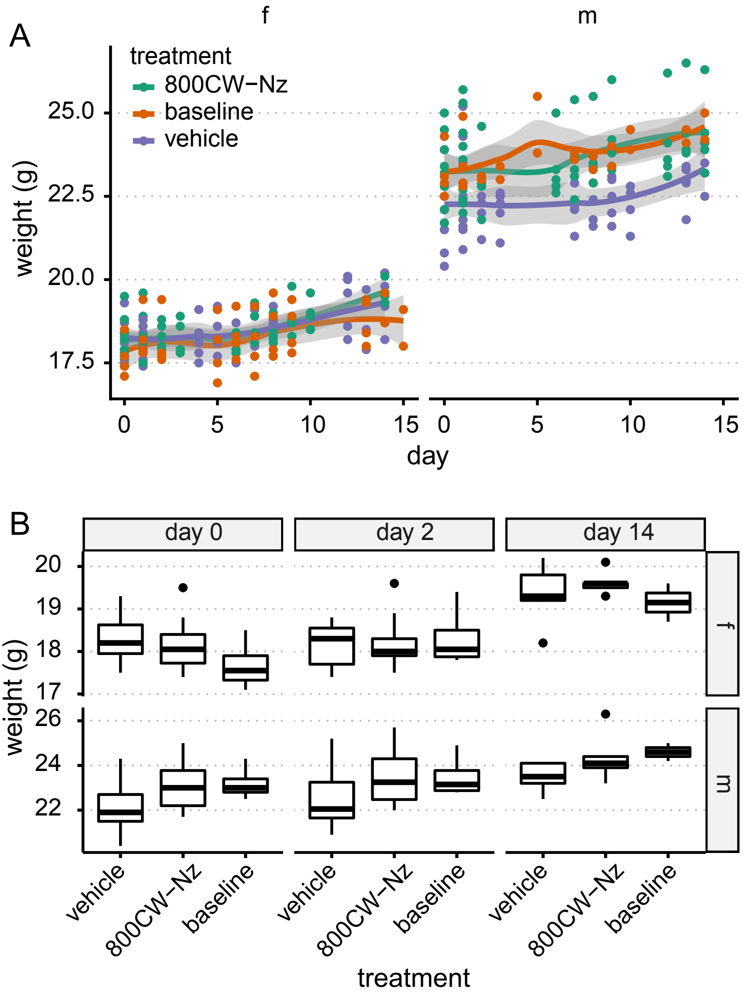

Mouse weights from toxicity studies.

A) Female (f) and male (m) mice were weighed 5 times a week during quarantine and the IRDye800CW-nimotzumab (800CW-Nz) toxicity study. B) Mouse weights at day 0, day 1, and day 14. In box plots hinges correspond to the first and third quartiles; whiskers extend from the hinge to the largest value no longer than 1.5 x the interquartile range.
