# Supplementary material for: Pre-clinical study of IRDye800CW-nimotuzumab formulation, stability, pharmacokinetics, and safety
Source: BMC Cancer. 2021 Mar 12;21:270. doi: 10.1186/s12885-021-08003-3 (PMC7953729; doi:10.1186/s12885-021-08003-3)
Supplement: Supplementary file 4 — Additional file 4. Weight of mouse liver, kidney, and spleen from toxicity studies. Graphs showing the weights of the liver, spleen and kidney of the mice used for the toxicity experiments. [file 12885_2021_8003_MOESM4_ESM.pdf]

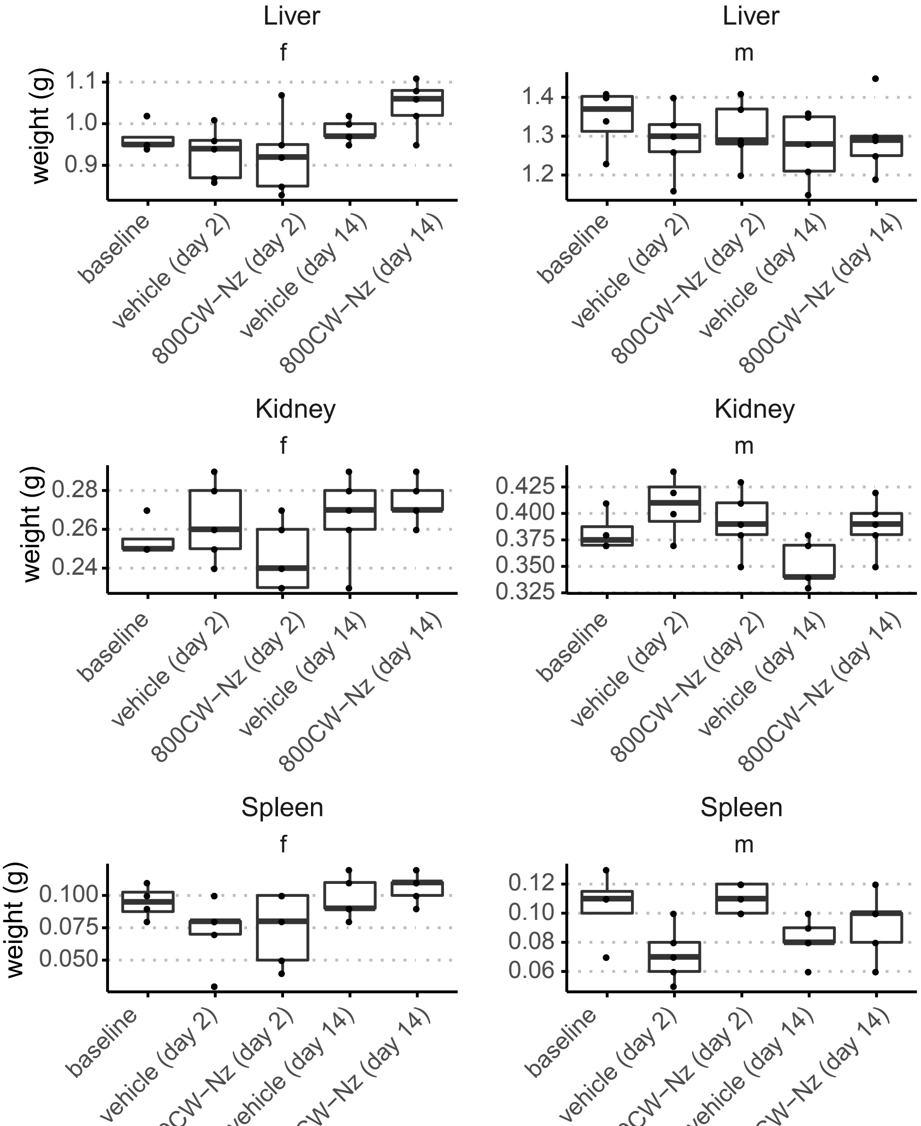

Weight of mouse liver, kidney, and spleen from toxicity studies
Weights of liver, kidney, and spleen were taken of the male (m) and female (f) mice at necropsy for the IRDye800CW-nimotuzumab (800CW-Nz) toxicity studies. In box plots hinges correspond to the first and third quartiles; whiskers extend from the hinge to the largest value no longer than 1.5 x the interquartile range.
