# Supplementary material for: Pre-clinical study of IRDye800CW-nimotuzumab formulation, stability, pharmacokinetics, and safety
Source: BMC Cancer. 2021 Mar 12;21:270. doi: 10.1186/s12885-021-08003-3 (PMC7953729; doi:10.1186/s12885-021-08003-3)
Supplement: Supplementary file 5 — Additional file 5. Clinical chemistry results from the toxicity study. Sodium, potassium, sodium: potassium (Na:K) ratio, chloride, calcium, phosphorus magnesium, urea, creatinine, amylase, lipase, glucose, cholesterol, bilirubin, alkaline phosphatase (Alk Phos), alanine aminotransferase (ALT), glutamate dehydrogenase (GLDH), CK (creatine kinase), protein, albumin, globulin and albumin:globulin (A:G) ratio measurements from the IRDye800CW-nimotuzumab (800CW-Nz) toxicity studies. [file 12885_2021_8003_MOESM5_ESM.pdf]

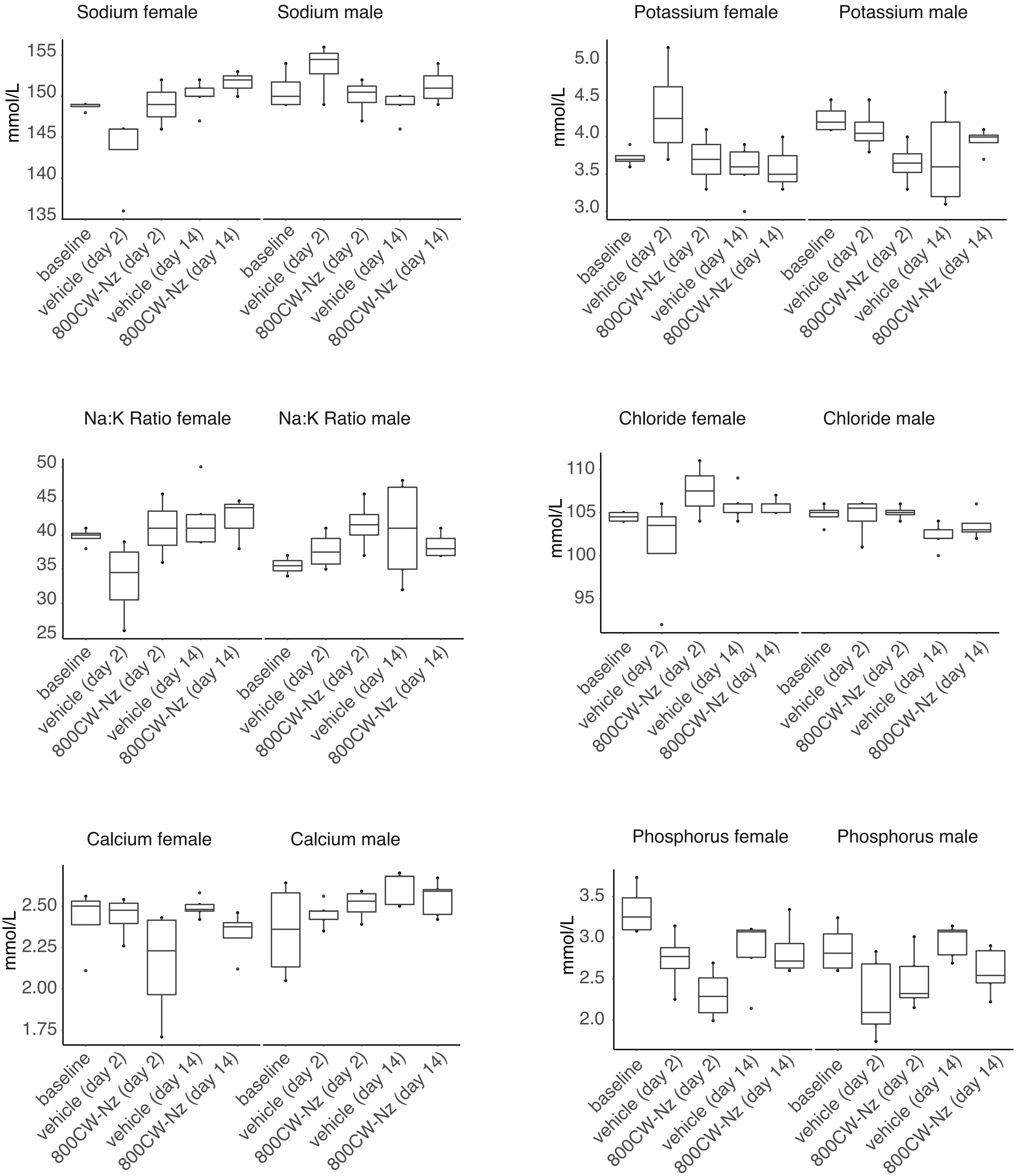
Sodium female
Sodium male
mmol/L
155
150
145
140
135
Potassium female
Potassium male
5.0
4.5
4.0
3.5
3.0
Na:K Ratio female
Na:K Ratio male
50
45
40
35
30
25
Chloride female
Chloride male
110
105
100
95
Calcium female
Calcium male
2.50
2.25
2.00
1.75
Phosphorus female
Phosphorus male
3.5
3.0
2.5
2.0
baseline
vehicle (day 2)
800CW-Nz (day 2)
vehicle (day 14)
800CW-Nz (day 14)

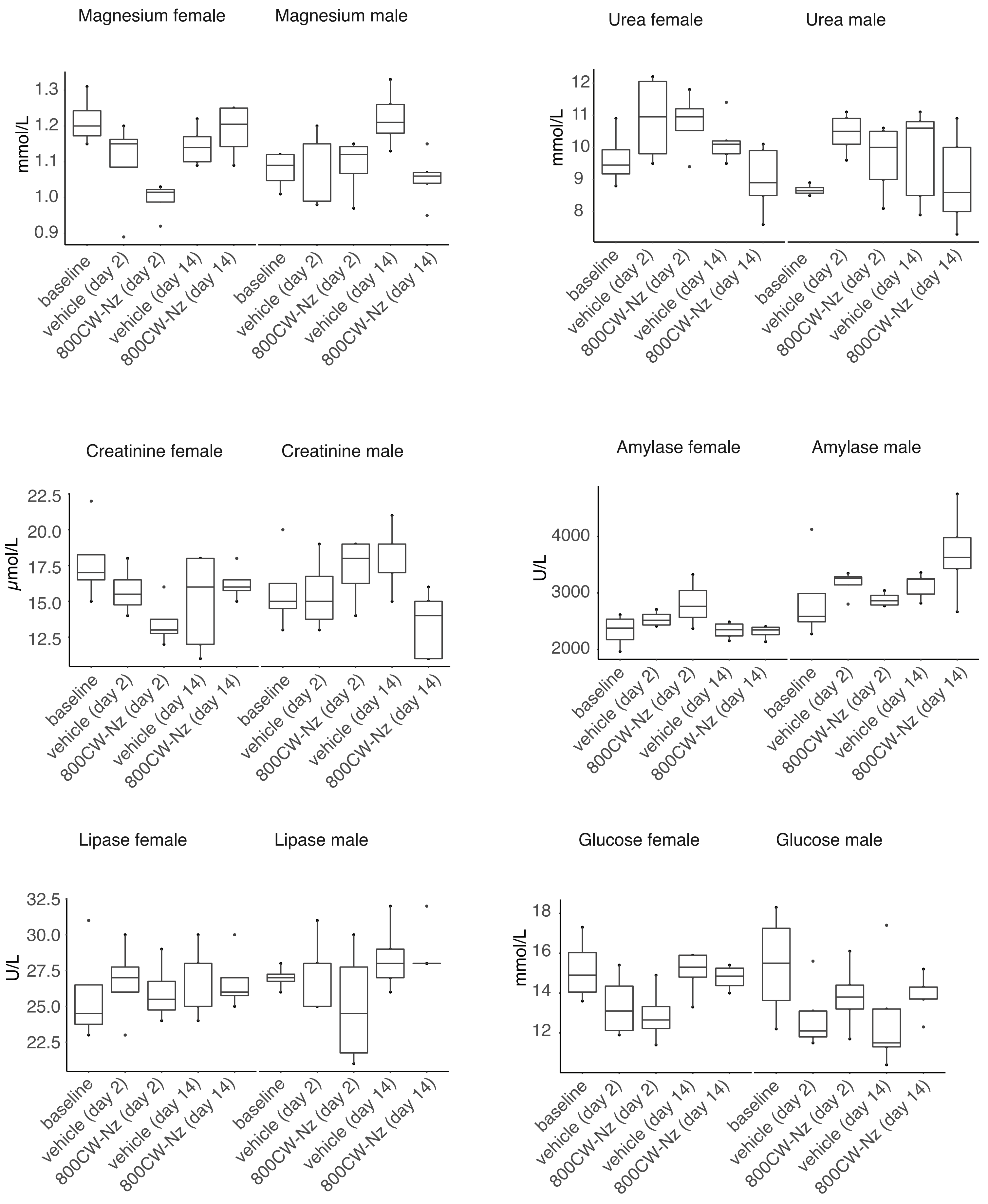

Magnesium female
Magnesium male
mmol/L
1.3
1.2
1.1
1.0
0.9
Urea female
Urea male
mmol/L
12
11
10
9
8
Creatinine female
Creatinine male
μmol/L
22.5
20.0
17.5
15.0
12.5
Amylase female
Amylase male
U/L
4000
3000
2000
Lipase female
Lipase male
U/L
32.5
30.0
27.5
25.0
22.5
Glucose female
Glucose male
mmol/L
18
16
14
12
baseline
vehicle (day 2)
800CW-Nz (day 2)
vehicle (day 14)
800CW-Nz (day 14)

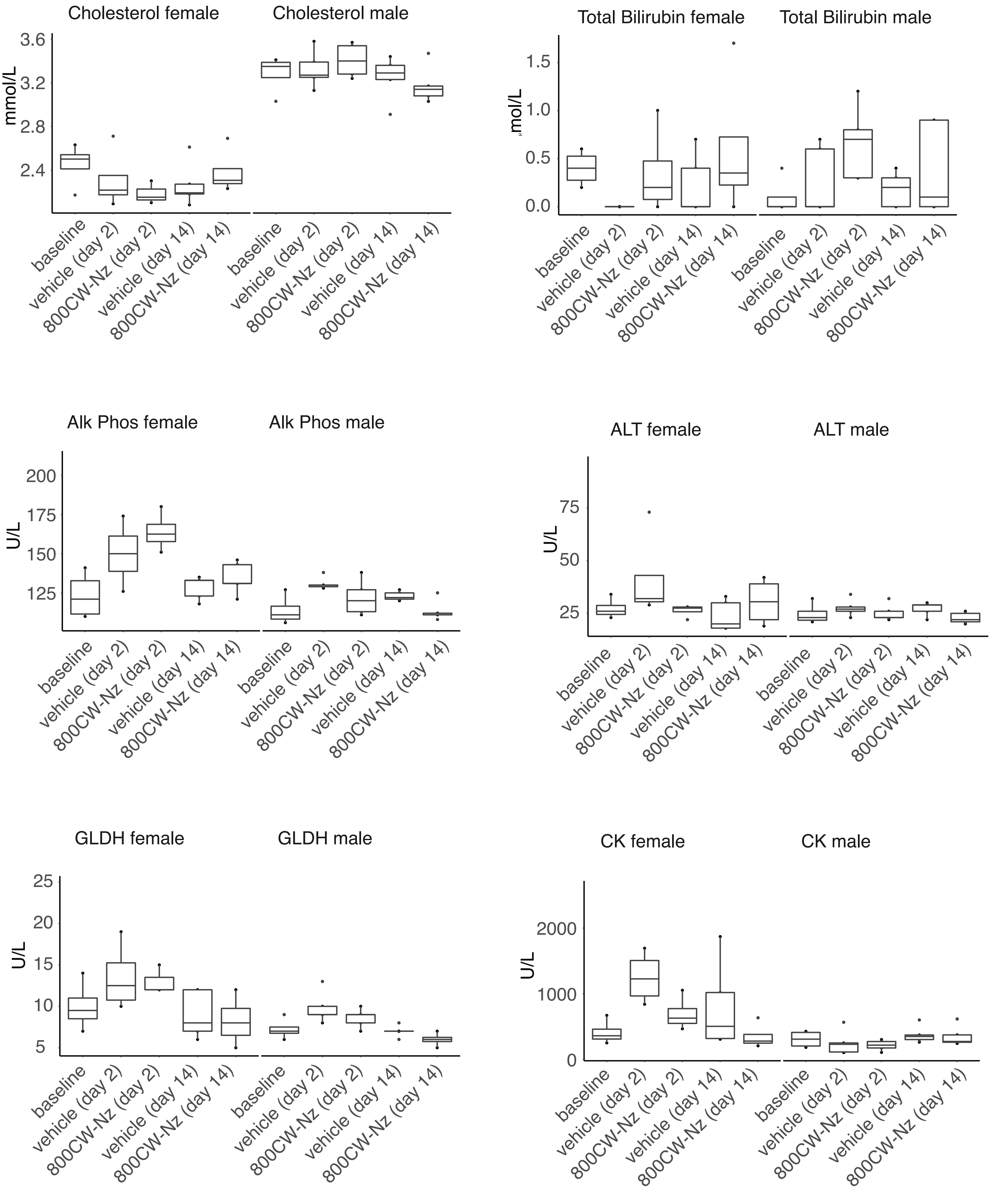

Cholesterol female
Cholesterol male
mmol/L
3.6
3.2
2.8
2.4
Total Bilirubin female
Total Bilirubin male
µmol/L
1.5
1.0
0.5
0.0
Alk Phos female
Alk Phos male
U/L
200
175
150
125
ALT female
ALT male
U/L
75
50
25
GLDH female
GLDH male
U/L
25
20
15
10
5
CK female
CK male
U/L
2000
1000
0
baseline
vehicle (day 2)
800CW-Nz (day 2)
vehicle (day 14)
800CW-Nz (day 14)

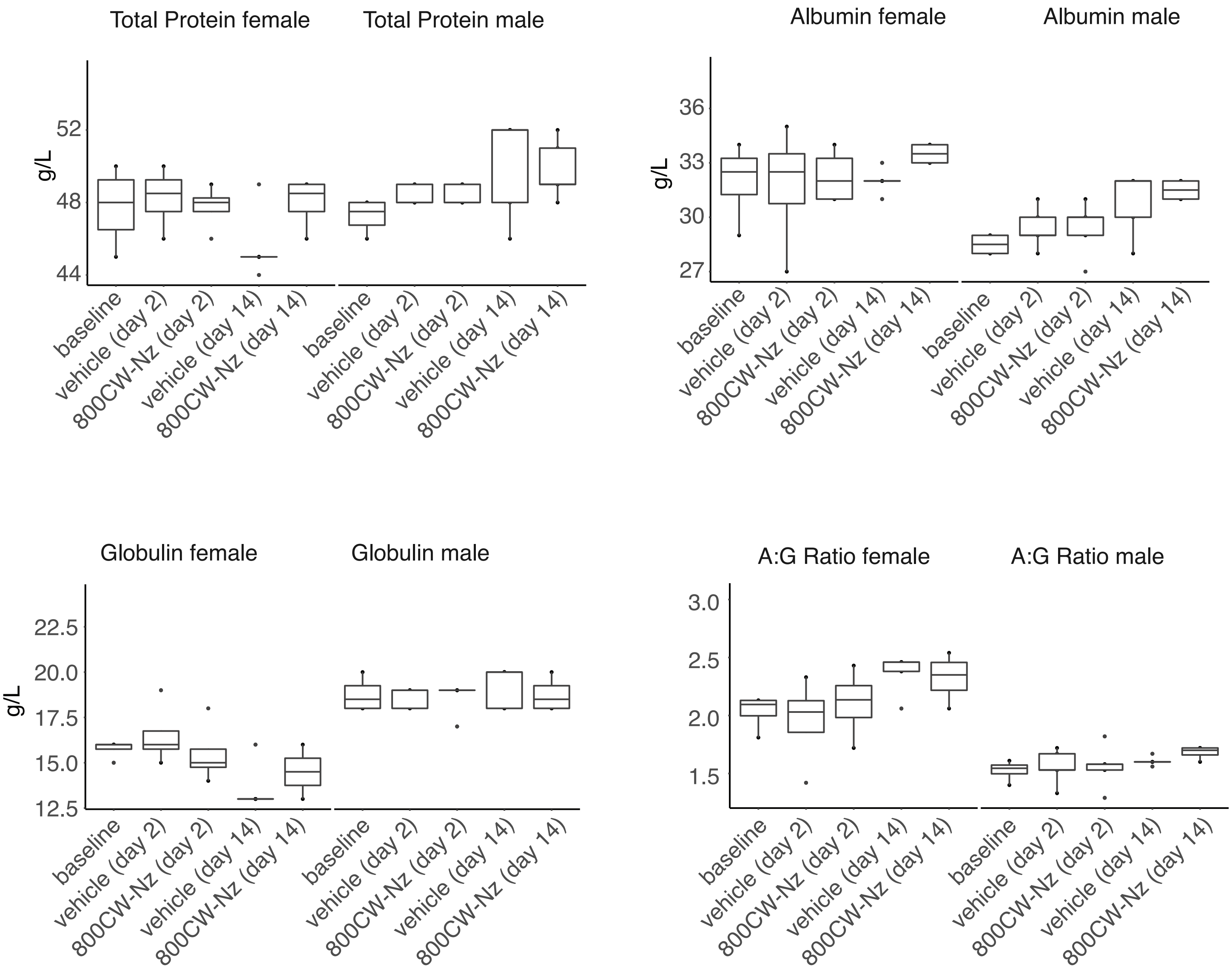

Clinical chemistry results from the toxicity study

Sodium, postassium, sodium:potassium (Na:K) ratio, chloride, calcium, phosphorus magnesium, urea, creatinine, amylase, lipase, glucose, cholesterol, bilirubin, alkaline phosphatase (Alk Phos), alanine aminotransferase (ALT), glutamate dehydrogenase (GLDH), CK (creatine kinase), protein, albumin, globulin and albumin:globulin (A:G) ratio measurements from the IRDye800CW-nimotuzumab (800CW-Nz) toxicity studies. In box plots hinges correspond to the first and third quartiles; whiskers extend from the hinge to the largest value no longer than 1.5 x the interquartile range.
