# Supplementary material for: Pre-clinical study of IRDye800CW-nimotuzumab formulation, stability, pharmacokinetics, and safety
Source: BMC Cancer. 2021 Mar 12;21:270. doi: 10.1186/s12885-021-08003-3 (PMC7953729; doi:10.1186/s12885-021-08003-3)
Supplement: Supplementary file 6 — Additional file 6. Hematology results from toxicity study. White blood cells (WBC), lymphocytes (LY), monocytes (MO), granulocytes (GR), red blood cells (RBC) and red blood cell distribution width (RDW), hematocrits (Hct), platelets (Plt), haemoglobin (Hgb), mean corpuscular haemoglobin (MCH), mean corpuscular haemoglobin count (MCHC) and mean corpuscular volume (MCV) measurements from the IRDye800CW-nimotuzumab (800CW-Nz) toxicity studies. [file 12885_2021_8003_MOESM6_ESM.pdf]

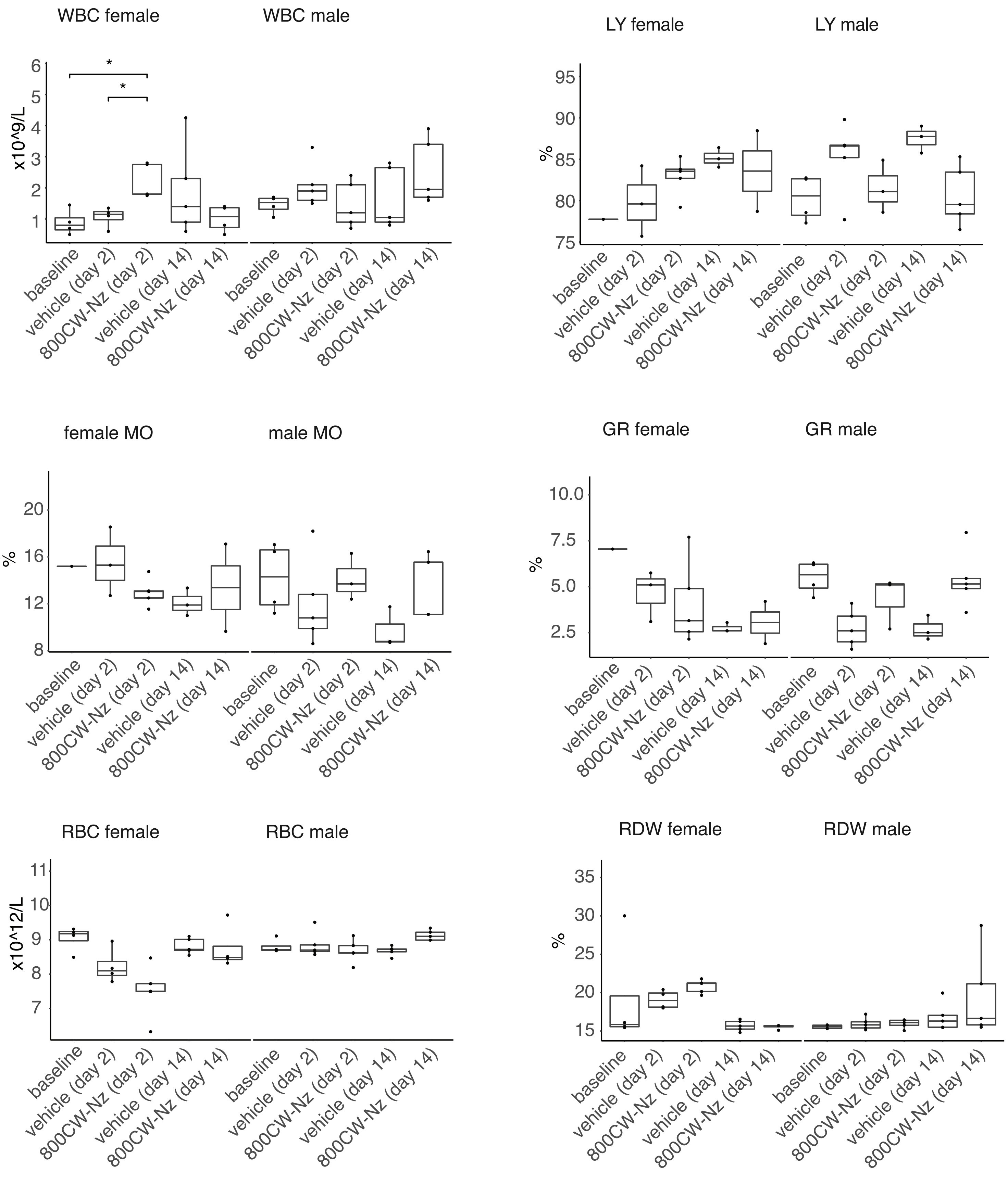
WBC female
WBC male
x10^9/L
1
2
3
4
5
6
*
*
LY female
LY male
%
75
80
85
90
95
female MO
male MO
%
8
12
16
20
GR female
GR male
%
2.5
5.0
7.5
10.0
RBC female
RBC male
x10^12/L
7
8
9
10
11
RDW female
RDW male
%
15
20
25
30
35
baseline
vehicle (day 2)
800CW-Nz (day 2)
vehicle (day 14)
800CW-Nz (day 14)

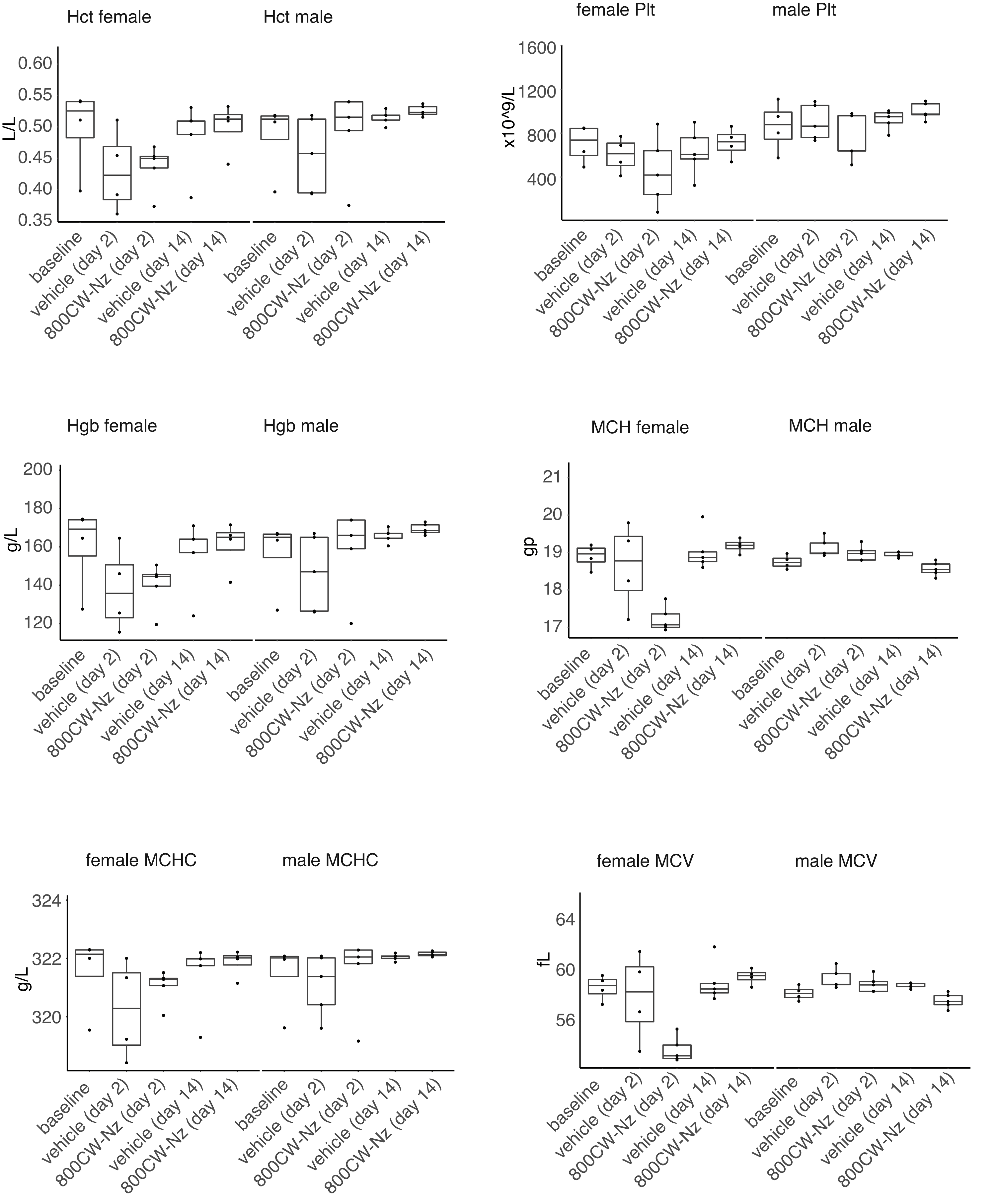

Hematology results from toxicity study

White blood cells (WBC), lymphocytes (LY), monocytes (MO), granulocytes (GR), red blood cells (RBC) and red blood cell distribution width (RDW), hematocrits (Hct), platelets (Plt), haemoglobin (Hgb), mean corpuscular haemoglobin (MCH), mean corpuscular haemoglobin count (MCHC) and mean corpuscular volume (MCV) measurements from the IRDye800CW-nimotuzumab (800CW-Nz) toxicity studies. In box plots hinges correspond to the first and third quartiles; whiskers extend from the hinge to the largest value no longer than 1.5 x the interquartile range.
