# Supplementary material for: Pre-clinical study of IRDye800CW-nimotuzumab formulation, stability, pharmacokinetics, and safety
Source: BMC Cancer. 2021 Mar 12;21:270. doi: 10.1186/s12885-021-08003-3 (PMC7953729; doi:10.1186/s12885-021-08003-3)
Supplement: Supplementary file 7 — Additional file 7. Table 2: Clinical trials on clinicaltrials.gov accessed 20,190,422 search term: “800CW OR IRDYE800CW OR IRDYE”. [file 12885_2021_8003_MOESM7_ESM.pdf]

Additional File 7

Clinical trials on clinicaltrials.gov accessed 20190424 search term: "800CW OR IRDYE800CW OR IRDYE"

nct_id	official_title
NCT03913806	FLuoresence Image Guided Surgery With A VEGF-targeted Tracer in Soft-tissue Sarcomas in Humans (FLASH)- A Feasibility Dose Escalation Study
NCT03877601	A Prospective Follow-up Intervention Study: Detection of Early Esophageal Cancer by Near-infrared Fluoresence Molecular Endoscopy Using Bevacizumab-800CW
NCT03925285	Image Guided Surgery in Sinonasal Inverted Papilloma by Targeting Vascular Endothelial Growth Factor
NCT03620292	Cholangiocarcinoma Detection Using an Intraoperative Fluorescence Image Guided Approach With Bevacizumab-IRDye 800CW
NCT03699332	A Phase I/II Study to Evaluate the Safety and Feasibility of Multi-modality Imaging Using Indium-111-DOTA-labetuzumab-IRDye800CW in Patients With Peritoneal Carcinomatosis of Colorectal Origin
NCT03582124	A Phase I/II Study Evaluating the Safety and Pharmacokinetics of Panitumumab-IRDye800 as an Optical Imaging Agent to Detect Lung Cancer During Surgical Procedures
NCT03407781	IRDye800CW-BBN PET-NIRF in Lower Grade Glioma
NCT03558724	Fluorescence Molecular Endoscopy of Locally Advanced Esophageal Carcinoma Using Bevacizumab-800CW to Evaluate Dose Response After Neoadjuvant Chemoradiotherapy: a Single-center Feasibility Study.
NCT03384238	A Phase I/II Study Evaluating the Safety and Pharmacokinetics of Panitumumab-IRDye800 as an Optical Imaging Agent to Detect Pancreas Cancer During Surgical Procedures
NCT03405142	A Phase II Study Evaluating Panitumumab-IRDye800 vs. Sentinel Node Biopsy and (Selective) Neck Dissection for Metastatic Lymph Node Identification in Patients With Head and Neck Cancer
NCT03134846	Image Guided Surgery for Margin Assessment of Head and Neck Cancer Using Cetuximab-IRDye800CW cONjugate
NCT02910804	PET-NIRF Dual Modality Imaging Guiding Surgery in Patients With Glioblastoma
NCT03643068	Phase 1A Study of the Safety of KSP/QRH Dimer in Humans for the Early Detection of GI Malignancies
NCT02855086	Open-Label Study Evaluating Cetuximab-IRDye800 as an Optical Imaging Agent to Detect Neoplasms During Neurosurgical Procedures
NCT02736578	Intraoperative Pancreatic Cancer Detection Using Multimodality Molecular Imaging
NCT03510208	Phase I/II, Open-Label Study Evaluating the Efficacy and Pharmacokinetics of Panitumumab-IRDye800 as an Optical Imaging Agent to Detect Neoplasms During Neurosurgical Procedures

NCT02583568	Phase II, Open-Label Study to Evaluate Safety and Explore Efficacy of Escalating Doses of Bevacizumab-IRDye800CW as an Optical Imaging Agent to Detect Cancer Tissue Delineation During Tumor Resection Surgery in Patients With Breast Cancer
NCT02743975	Intraoperative Detection of Cancer Tissue in Pancreatic Adenocarcinoma Using a VEGF-targeted Optical Fluorescent Imaging Tracer, A Multicentre Feasibility Dose Escalation Study
NCT02497599	A Phase 1 Study to Evaluate the Safety and Feasibility of Intraoperative Detection of Clear Cell Renal Cell Carcinoma Using Indium-111-DOTA-girentuximab-IRDye800CW
NCT03733210	Pilot Study Evaluating Panitumumab-IRDye800 and 89Zr-Panitumumab for Dual-Modality Imaging for Nodal Staging in Head and Neck Cancer
NCT02415881	Phase I, Open-label Study Evaluating the Safety and Pharmacokinetics of Escalating Doses of Panitumumab-IRDye800 as an Optical Imaging Agent to Detect Head and Neck Cancer During Surgical Procedures
NCT02975219	Determine the Feasibility of Detecting Endometriosis During Surgery Using a Molecular Targeted Fluorescent Imaging Tracer
NCT02113202	Visualization of a VEGF-targeted Near-Infrared Fluorescent Tracer in Patients With Familial Adenomatous Polyposis During Fluorescence Endoscopy A Single Center Pilot Intervention Study
NCT02129933	A Pilot Intervention Study for the Use of VEGF-targeted Fluorescence Near-Infrared (NIR) Endoscopy in (Pre)Malignant Esophageal Lesions
NCT01987375	Phase I, Open-label Study Evaluating the Safety and Pharmacokinetics of Escalating Doses of Cetuximab-IRDye800 as an Optical Imaging Agent to Detect Cancer During Surgical Procedures
NCT01972373	Visualization of a VEGF-targeted Optical Fluorescent Imaging Tracer in Rectal Cancer During Flexible NIR Fluorescence Endoscopy
NCT01508572	Validation of Uptake of a VEGF-targeted Optical Fluorescent Imaging Tracer in Surgical Specimens of Breast Cancer and Application of Pre- and Intra-operative Human Molecular Fluorescence Imaging Techniques. A Multicenter Feasibility Study
NCT03161418	Study of the Safety of KSP Heptapeptide (KSP-910638G) in Humans for the Early Detection of GI Malignancies
NCT03923881	Multispectral Optoacoustic Imaging Using Cetuximab-800CW for Detection of Cervical Lymph Node Metastases: a Single Center Proof of Concept Study
NCT03757507	Investigation of Plaque Instability: Identification of Atherosclerotic Plaque Angiogenesis Using Bevacizumab-800CW and Optoacoustic Imaging: a Single Center Proof of Concept Study (CAROTID-OPTOLIGHT)
NCT03282461	A Phase 0 Open Label, Single-center Clinical Trial of ABY-029, an Anti-EGFR Fluorescence Imaging Agent Via Single Intravenous Injection to Subjects With Operable Head and Neck Cancer.
NCT03154411	A Phase 0 Open Label, Single-center Clinical Trial of ABY-029, an Anti-EGFR Fluorescence Imaging Agent Via Single Intravenous

	Injection to Subjects With Primary Sarcoma.
NCT02901925	A Phase 0 Open Label, Single-center Clinical Trial of ABY-029, an Anti-EGFR Fluorescence Imaging Agent Via Single Intravenous Injection to Subjects With Recurrent Glioma
NCT03852576	Phase 1B In-vivo Study of KSP/QRH Heptapeptide Dimer for Detection of Neoplasia in the Esophagus
