# Supplementary material for: Pre-clinical study of IRDye800CW-nimotuzumab formulation, stability, pharmacokinetics, and safety
Source: BMC Cancer. 2021 Mar 12;21:270. doi: 10.1186/s12885-021-08003-3 (PMC7953729; doi:10.1186/s12885-021-08003-3)
Supplement: Supplementary file 8 — Additional file 8. Indications of clinical trials with IRDye800CW. A table listing the clinical trials, indications and the number of indications (n). [file 12885_2021_8003_MOESM8_ESM.pdf]

Additional File 8

Indications of clinical trials.

Indication mapped	n	NCT IDs
head and neck	8	NCT02415881, NCT01987375, NCT03282461, NCT03405142, NCT03134846, NCT03925285, NCT03923881, NCT03733210
brain	5	NCT02855086, NCT02910804, NCT03407781, NCT02901925, NCT03510208
esophageal	4	NCT03877601, NCT03852576, NCT03558724, NCT02129933
pancreatic	3	NCT03384238, NCT02736578, NCT02743975
breast	2	NCT02583568, NCT01508572
colon	2	NCT02113202, NCT03699332
healthy	2	NCT03643068, NCT03161418
sarcoma	2	NCT03154411, NCT03913806
atherosclerosis	1	NCT03757507
endrometriosis	1	NCT02975219
liver	1	NCT03620292
lung	1	NCT03582124
rectal	1	NCT01972373
renal	1	NCT02497599
