# Supplementary material for: Pre-clinical study of IRDye800CW-nimotuzumab formulation, stability, pharmacokinetics, and safety
Source: BMC Cancer. 2021 Mar 12;21:270. doi: 10.1186/s12885-021-08003-3 (PMC7953729; doi:10.1186/s12885-021-08003-3)
Supplement: Supplementary file 9 — Additional file 9. Clinical trials by probe and trial status. A table listing clinical trials by probe of probes similar to IRDye800CW-nimotuzumab showing trials ‘completed’, ‘in progress’ and ‘terminated’ or ‘withdrawn’. [file 12885_2021_8003_MOESM9_ESM.pdf]

Additional File 9

Clinical trials by probe and trial status.

Parent	conjugate	Complete	In-progress	terminated or withdrawn	unknown	total
Bevacizumab	IRDye800CW	5	8	-	-	13
Panitumumab	IRDye800CW	-	6	-	-	6
Cetuximab	IRDye800CW	-	2	3	-	5
ABY-029	IRDye800CW	-	3	-	-	3
KSP*	IRDye800CW	2	1	-	-	3
BBN	IRDye800CW/68Ga	-	1	-	1	2
Girentuximab	IRDye800CW/Indium-111-DOTA	-	1	-	-	1
Labetuzumab	IRDye800CW/Indium-111-DOTA	-	1	-	-	1

**KSP indicates one of three probes: KSP-QRH-E3, KSP-910638G, or KSP/QRH dimer*
